# Validated Synthetic Patient Generation for Small Longitudinal Cohorts: Coagulation Dynamics Across Pregnancy

**Published:** 2026-08-11

**Authors:** Jeffrey D. Varner, Maria Cristina Bravo, Carole McBride, Thomas Orfeo, Ira Bernstein

**Affiliations:** 1 Robert F. Smith School of Chemical and Biomolecular Engineering, Cornell University, Ithaca, NY 14850; 2 Department of Biochemistry, The Robert Larner, M.D. College of Medicine, University of Vermont Medical Center, Burlington, VT 05401; 3 Department of Obstetrics, Gynecology, and Reproductive Sciences, The Robert Larner, M.D. College of Medicine, University of Vermont Medical Center, Burlington, VT 05401

**Keywords:** synthetic data generation, stochastic attention, modern Hopfield networks, multiplicity weighting, coagulation, pregnancy, longitudinal data, mechanistic validation

## Abstract

Small longitudinal cohorts, common in maternal health, rare diseases, and early-phase trials, limit computational modeling because enrollment is slow and the data are too sparse to train reliable models. We present multiplicity-weighted Stochastic Attention (SA), a generative framework based on modern Hopfield networks. Stochastic attention stores real patient profiles as memory patterns in a continuous energy landscape. The resulting distribution is a finite mixture with one component centered on each stored profile. We generated the reported cohort with Langevin dynamics; direct sampling from the same distribution reproduced its fidelity and membership-inference results. Multiplicity weights amplify selected subgroups at inference time without retraining. We applied the method to longitudinal coagulation data from 23 patients, with 72 features measured before pregnancy and during the first and third trimesters. The cohort included three patients with polycystic ovary syndrome and five who developed preeclampsia. Across statistical, structural, and mechanistic tests, including a separately specified coagulation model that was blind to the generator but calibrated on the same cohort, the synthetic profiles closely matched the real profiles under the measures applied at this sample size. A model calibrated on synthetic profiles predicted held-out real-patient TGA measurements as well as one calibrated on real profiles. These results provide proof of concept for using SA in the tested modeling tasks, including mechanistic calibration and hypothesis generation, with very small longitudinal cohorts.

## 1 Introduction

In 2021, the United States maternal mortality rate reached 32.9 deaths per 100,000 live births, the highest level since 1965 [Toy, 2023, Harris, 2023]. Between 2018 and 2022, Non-Hispanic Black women died at 2.8 times the rate of White women, and disorders related to pregnancy, including hemorrhage and venous complications, were the leading underlying cause of death, accounting for 17.4% of 6,283 pregnancy-related deaths [Chen et al., 2025]. These figures highlight the role of the coagulation system in maternal outcomes. Blood coagulation is a complex enzymatic cascade in which a small tissue factor (TF) stimulus triggers a series of serine protease activation reactions that ultimately produce thrombin, the central effector of hemostasis [Mann, 2011, Butenas et al., 2009]. Thrombin cleaves fibrinogen into fibrin, activates platelets, and amplifies its own production through positive feedback via Factors V, VIII, and XI, while simultaneously initiating its own shutdown through the thrombomodulin–protein C anticoagulant pathway [Hockin et al., 2002, Bravo et al., 2012]. Pregnancy alters this hemostatic balance, shifting toward a hypercoagulable state that prepares for delivery [Hellgren, 2003, Grouzi et al., 2022, Bremme, 2003]. Procoagulant factors including fibrinogen, Factor VIII, and von Willebrand factor increase substantially across gestation, while natural anticoagulants such as antithrombin and protein S decline. Two conditions further shift this balance in ways that are not fully understood. Polycystic ovary syndrome (PCOS), a hormonal and metabolic disorder affecting approximately 6.6% of reproductive-age women [Azziz et al., 2004], has been associated with impaired fibrinolysis and a prothrombotic tendency [Palomba et al., 2015]. Preeclampsia (PE), a hypertensive disorder that complicates 2–8% of pregnancies worldwide [Abalos et al., 2014], is characterized by endothelial dysfunction, platelet activation, and altered thrombin generation [Dusse et al., 2011]. Mathematical models of the coagulation cascade have provided mechanistic insight into patient-specific thrombin generation [Luan et al., 2007, 2010]. These range from the original Hockin–Mann stoichiometric framework [Hockin et al., 2002] through extensions incorporating the thrombomodulin–protein C pathway [Bravo et al., 2012] to the Brummel-Ziedins 2012 (BZ2012) model with detailed activated protein C (APC)-mediated Factor Va inactivation kinetics [Brummel-Ziedins et al., 2012]. However, applying these models to pregnancy cohorts requires longitudinal datasets capturing coagulation factors, inhibitors, and thrombin generation assay (TGA) measurements across gestation, data that is rare and expensive to collect.

The core barrier is sample size. Longitudinal pregnancy coagulation studies typically enroll tens of patients with complete follow-up across multiple trimesters, yielding datasets where the number of features p (coagulation factors, TGA parameters, viscoelastic measurements) exceeds the number of patients n. Rare complications compound this problem. Small longitudinal cohorts inevitably contain only a handful of patients with any given condition, far too few for condition-specific statistical analysis. This n<p regime limits conventional approaches because covariance matrices are rank-deficient, cross-validation is unreliable, and overfitting is nearly inevitable [Hastie et al., 2009]. Synthetic data can provide dense simulated inputs for mechanistic modeling, workflow testing, and hypothesis generation [Gonzales et al., 2023, Kühnel et al., 2024]. A further motivation was to support the development of deep learning models. However, existing methods face limitations in the small-cohort longitudinal setting. Sampling from a fitted multivariate normal (MVN) distribution is singular when n<p and must be regularized [Ledoit and Wolf, 2004], introducing bias that distorts the joint distribution. The multivariate-normal model also assumes Gaussian marginals and linear correlations and cannot amplify a specific subpopulation while preserving its signatures. More expressive models such as generative adversarial networks (GANs) and variational autoencoders (VAEs) [Goodfellow et al., 2014, Kingma and Welling, 2014, Xu et al., 2019], including recent longitudinal extensions [Kühnel et al., 2024], require substantially larger training sets and are prone to mode collapse at small n [De Mitri et al., 2025] (we confirm this empirically for Conditional Tabular GAN (CTGAN) at n=23 in this work). A generative method is needed that can operate directly on the geometry of a small dataset without estimating a full covariance model.

Stochastic Attention (SA) is such a framework. Rather than fitting a full covariance model or training a neural generator, SA treats the stored patient profiles themselves as the model. Building on modern Hopfield network theory [Ramsauer et al., 2021], each profile becomes a memory pattern in a continuous energy landscape. This energy induces a continuous probability distribution that is exactly a finite Gaussian mixture in the reduced space, with one nonzero-variance component centered on each stored pattern. Sampling from this distribution therefore produces continuous variations around the stored patterns rather than exact copies. Because sampling occurs in a principal component analysis (PCA)-reduced linear subspace anchored on the observed cohort, SA never forms a full p×p covariance matrix and so avoids both the rank deficiency that limits MVN and the mode collapse that limits GAN/VAE approaches when n<p. We recently introduced a multiplicity-weighted extension of the Hopfield energy that attaches a per-pattern weight to each stored profile [Varner, 2026a], which allows continuous interpolation between unconditioned generation and targeted amplification of a rare subpopulation at inference time, without retraining. This study asked whether SA could faithfully generate *longitudinal* trajectories from a small cohort. In earlier proceedings work, we paired our mechanistic coagulation pipeline with per-visit MVN sampling to produce synthetic populations [Bravo et al., 2023]; that approach captured single-visit marginals but, by fitting each visit independently, generated patients whose trajectories across gestation were statistically incoherent. Small longitudinal cohorts require a generator that preserves this joint structure.

In this study, we used multiplicity-weighted SA to generate complete longitudinal patient profiles from a small pregnancy coagulation cohort (K=23 patients, 72 features per visit, 3 visits) that includes rare clinical subgroups (PCOS, n=3; Developed PE, n=5). Our pipeline concatenates each patient’s multi-visit profile into a single vector, applies PCA for dimensionality reduction, and generates new patients via multiplicity-weighted Langevin dynamics on the Hopfield energy landscape, with a direction-magnitude decomposition that preserves the natural variance structure of continuous clinical measurements. We then asked whether the synthetic patients reproduced marginal feature distributions, the cross-visit covariance structure, condition-specific signatures of rare clinical subgroups, and the biological consistency obtained when the profiles were processed by a separately specified, generator-blind ordinary differential equation (ODE) model of the coagulation cascade that was calibrated on the same real cohort [Brummel-Ziedins et al., 2012]. As a downstream-utility test, we asked whether a mechanistic model calibrated entirely on synthetic patients could predict held-out real-patient TGA measurements. As a proof of concept, the results show that SA recovered the statistical structure of this cohort and matched its mechanistic plausibility under the tests used here. Generalization to other clinical cohorts remains to be tested.

## 2 Results

We generated N=100 synthetic longitudinal patient profiles using SA and compared them against the K=23 real patients across four validation levels. As a baseline, we generated N=100 patients from a regularized multivariate normal (MVN) distribution fitted to the same data.

### 2.1 Marginal Plausibility

We first assessed whether SA-generated patients reproduced per-feature summary statistics across all three visits. The pooled median mean relative error (MRE) across all 216 feature–visit entries was 1.2% (95% bootstrap CI: 1.0–1.6%), with 193 of 216 entries (89%) below 5% (Table S5), indicating that SA captured the central tendencies of the real cohort. Per-visit MREs for representative coagulation features are summarized in Table 2, where Factor II and Factor VIII fell below 2%, antithrombin below 3%, and fibrinogen below 1% across all three visits. Marginal agreement alone could result from profiles that closely copied stored records, so we also quantified similarity to stored profiles with two metrics (Table S9). The mean novelty score $\left(1-\max_{k}\;\cos\left(\hat{\xi },{\hat{m}}_{k}\right)\right)$ was 0.50 at $\beta =\beta^{*}$. The median nearest-neighbor distance from each synthetic patient to its closest real patient was 6.14 standardized units, compared with 6.87 for the median real-to-real nearest-neighbor distance, a ratio of 0.89 indicating that synthetic patients were approximately as far from real patients as real patients were from each other. Thus, stochastic attention matched the central tendencies without reproducing stored profiles exactly.

We next examined whether known physiological relationships and longitudinal trajectories were preserved in the synthetic cohort. Pairwise scatter plots of coagulation factor levels against thrombin generation parameters showed that SA-generated patients occupied the same joint regions as real patients (Fig. 1). The expected inverse relationship between antithrombin and TF-initiated peak thrombin was preserved, as were the positive associations between Factor VIII and peak, prothrombin and endogenous thrombin potential (ETP), and the inverse relationship between antithrombin and ETP. Per-visit means and standard deviations for six key coagulation features showed that synthetic patients tracked the real longitudinal trajectories closely, with overlapping confidence bands at all three visits (Fig. 2). Fibrinogen, Factor VIII, and von Willebrand factor showed the expected monotonic increases from baseline through the third trimester in both cohorts, while Factor II increased modestly and antithrombin declined then partially recovered, consistent with the known hypercoagulable shift of pregnancy. The 72 features also included viscoelastic (ROTEM) and fibrinolytic parameters, which had comparable MREs to the coagulation factors (Table S5). These variables influenced the generated profiles through their loadings on the retained principal components. Together, these results showed that the synthetic cohort preserved the tested physiological relationships and the direction and magnitude of longitudinal change.

To determine whether standard deep tabular models provided a viable baseline at this sample size, we compared SA against Conditional Tabular GAN (CTGAN) and Tabular Variational Autoencoder (TVAE) [Xu et al., 2019] (Table S6). The CTGAN model produced median MREs of approximately 19% across all tested epoch counts (300–3,000), consistent with mode collapse at this sample size. The TVAE model achieved comparable marginal fidelity at high epoch counts (median MRE 1.8% at 3,000 epochs), but operated on per-visit rows (n=90) rather than concatenated longitudinal profiles (n=23), meaning it could not capture cross-visit covariance structure and had no mechanism for conditional subgroup generation. Thus, neither deep model provided a suitable complete-profile baseline for this 23-patient cohort.

To examine the role of the shared PCA representation, we compared stochastic attention with a fitted two-component diagonal-covariance Gaussian mixture, a kernel-density estimator, k-nearest-neighbor interpolation, and a Gaussian copula in the same d=18 PCA subspace (Supplementary Methods, PCA-space ablation; Supplementary Table S13). Nearest-neighbor samples remained close to stored profiles: median novelty, $1-\max_{k}\;\cos\left(\xi ,{\hat{m}}_{k}\right)$, was 0.05, and only 5% of the synthetic patients exceeded the 0.2 novelty cutoff. The kernel-density estimator had marginal error 1.9% and mechanistic overlap 0.82 (the fraction of pooled synthetic predicted-to-recorded TGA ratios within the real 5th–95th percentile range). The two-component mixture and copula matched stochastic attention more closely in marginal error (1.1% and 1.3% versus 1.2%) and mechanistic overlap (0.92 and 0.93 versus 0.90). All profiles from stochastic attention and the copula exceeded the novelty cutoff, compared with 94% from the two-component mixture. Stochastic attention had a larger empirical cross-visit correlation error than the two-component mixture and copula (0.64 versus 0.42 for both). This metric measured agreement with the correlation matrix of the same 23 patients used to construct the stochastic-attention memory matrix or fit the baseline generators; it therefore assessed in-sample reproduction rather than recovery of the unknown population correlation matrix. Holding the PCA representation constant showed that the marginal errors and mechanistic overlaps achieved by the two-component mixture and copula were similar to those of stochastic attention and therefore were not specific to the stochastic-attention generator. In contrast, empirical correlation error and novelty differed across generators. Thus, generator choice affected these aspects of performance, and no generator performed best on every measure.

### 2.2 Cross-Visit Covariance Structure

Having established that SA reproduces marginal distributions and pairwise relationships, we next asked whether it preserves the higher-order joint structure across visits. We compared the full 216 × 216 cross-visit correlation matrices for real, SA-generated, and MVN-generated populations. The real data exhibited a characteristic block structure with strong within-visit correlations along the diagonal and structured off-diagonal blocks reflecting cross-visit dependencies; for example, a patient’s Visit 1 Factor X level predicting their Visit 3 Factor X level. We found that SA preserved this block structure, including the off-diagonal cross-visit correlations (Fig. 3). The SA–Real residual matrix showed small, unstructured deviations distributed throughout, whereas the MVN–Real residual was notably smoother, reflecting the regularization-induced shrinkage of off-diagonal correlations toward zero. This difference was most apparent in the off-diagonal blocks, where MVN systematically underestimated cross-visit dependencies that SA retained. We note that Pearson correlation captures linear associations; Spearman rank correlations, used in the mechanistic validation, would additionally capture monotonic nonlinear dependencies. Within the linear analysis used here, stochastic attention retained the observed cross-visit structure more closely than the regularized MVN baseline.

We examined the eigenvalue spectrum of the sample covariance matrix to understand the structural origin of this difference (Fig. S2). The real data had rank 22, reflecting the K=23 patient constraint. Stochastic attention and MVN make different trade-offs in handling this rank deficiency: SA truncates via PCA (zeroing variance beyond component 18), while MVN regularizes via Ledoit–Wolf shrinkage (providing a full-rank estimate by inflating eigenvalues in the 194 null dimensions). The consequence of MVN’s approach is that it introduces variance in dimensions where the data contain no signal, which manifested as increased dispersion in PCA projections. Visit-specific PCA projections showed this dispersion gap (Supplementary Fig. S8). Patients generated by stochastic attention occupied the same region of PCA space as real patients across all three visits, while MVN-generated patients showed substantially greater scatter, particularly at Visits 2 and 3. The spectrum and projections therefore linked the MVN baseline’s greater scatter to variance added in directions unsupported by the cohort.

### 2.3 Robustness to Sample Size, Dimensionality, and Nonlinearity

Results from a single cohort of 23 patients could not show how stochastic attention would perform if the amount or structure of the available data changed. We therefore asked three questions in controlled tests (Methods, Sec. 5.5). First, could generated profiles recover the true covariance? We simulated datasets in which the covariance was known. We varied the size of the simulated datasets from 8 to 80 profiles, the number of variables per profile from 30 to 216, and the complexity of the correlation patterns. From each dataset, we constructed a stochastic-attention memory matrix. For comparison, we estimated a Gaussian generator from the dataset’s sample mean and covariance. Each generator then produced 100 profiles. We compared the covariance of each generated cohort with the known population covariance. Stochastic attention had lower error in 35 of the 39 settings in which the simulated dataset contained fewer profiles than variables (Fig. 4A). For simulations matching the clinical dataset in this study in terms of size, with 23 profiles and 216 variables per profile, the Gaussian generator’s covariance error was approximately 1.5 times the stochastic-attention error after averaging across the three levels of correlation-pattern complexity. Second, we examined how well stochastic attention preserved characteristics of the full 23-patient cohort when fewer patients were available. Using random subsets of 20, 15, 10, or 8 patients, we repeated the random selection ten times at each size, constructed a new memory matrix from each, generated 100 profiles, and compared every generated cohort with the same full 23-patient reference. As the subset size decreased from 20 to 8, error in the cross-visit correlations increased from 0.63 to 1.11 (Fig. 4B), the median error in variable means increased from 2.0% to 4.9%, and overlap with the mechanistic-model reference remained near 0.90. Third, could stochastic attention and the Gaussian generator reproduce curved relationships? We used a noisy S-curve, a simulated path that resembled the letter S because it had two opposing bends. We spread this path across either 30 or 120 variables while preserving its shape (Methods, Sec. 5.5). For each amount of curvature and each number of variables, we created five simulated datasets, each with 23 profiles. From every dataset, we constructed a stochastic-attention memory matrix and estimated a Gaussian generator; each generator produced 100 profiles. As curvature increased, stochastic-attention profiles moved farther from the curve. At curvature 0.5, their mean distance was at least twice the straight-line value after averaging across the five repeats and both numbers of variables. It remained below this threshold at curvature 0.25. Despite this decline, at every nonzero curvature, stochastic-attention profiles remained closer to the curve on average than Gaussian profiles. However, the Gaussian generator reproduced the population covariance more accurately than stochastic attention for the curved data. Together, these tests showed that stochastic attention recovered covariance more accurately than the Gaussian comparator in most of the small linear simulations with more variables than profiles, but its clinical correlations and variable means became less accurate when fewer patients were used. On curved data, each generator performed better on a different measure.

### 2.4 Interpolation and Extrapolation

The robustness tests examined recovery under controlled changes in the data. We next asked whether profiles generated from the clinical cohort remained within the real cohort or extended beyond it. Every synthetic profile lay outside the convex hull of the 23 real profiles in the d=18 PCA space (Supplementary Table S14). However, each real profile also lay outside the hull of the other 22, so binary membership was uninformative in this sparse space. We therefore compared hull distances against 22 real profiles. Each real calculation omitted the profile being evaluated, while each synthetic calculation omitted its nearest real profile. If generation extrapolated unusually far, synthetic distances would exceed leave-one-out real distances. The medians were 11.0 and 11.9, respectively, and a descriptive Mann–Whitney test did not detect a difference (*p*=0.07). While this did not establish equivalence, it provided no evidence that synthetic profiles extended farther from the cohort than real profiles treated as unseen. Geometric similarity did not establish biological plausibility, so we applied two further checks. The first required nonnegative values where appropriate and limited coagulation-factor and TGA values to five real-cohort standard deviations from the mean. Fifty-four of 100 synthetic profiles passed; the other 46 contained a negative estradiol or progesterone value. The unconstrained multivariate-normal baseline produced negative hormones in 43 profiles. Neither method enforced positivity, and positive factors in these draws did not guarantee positivity in others. The second check used the BZ2012 model to test the generated factor combinations. Estradiol and progesterone were not model inputs, and all nine coagulation factor inputs were positive, so all 100 profiles were included. For each TGA feature, 83–92% of profiles had a predicted-to-recorded ratio within the real 5th–95th percentile range. Forty-one met this criterion for every feature and visit, and 24 passed both checks. Positive-variable transformations or constrained decoding could prevent negative values. Together, these checks separated geometric extrapolation from biological plausibility. Hull distance provided no evidence that synthetic profiles extended farther than real profiles treated as unseen, but only 24 of 100 profiles passed both the biological and strict mechanistic criteria. Thus, hull position alone could not determine whether an individual generated profile was biologically plausible.

### 2.5 Membership-Inference Risk

Geometric proximity raised a separate privacy question: could a synthetic cohort reveal whether an already-known de-identified assay profile had been stored in the memory matrix? To answer this question, we tested whether distance from a candidate profile to the synthetic cohort could distinguish profiles used to construct the memory matrix from profiles withheld from it. In each of 40 random splits of the 23-patient cohort, we constructed the memory matrix from 15 profiles, held out the other 8, and generated a synthetic cohort. A smaller distance to the nearest synthetic profile provided stronger evidence that a candidate had been stored. Stored profiles were closer to the synthetic cohort than held-out profiles (median 10.7 versus 15.4; Supplementary Table S15). We quantified this discrimination using the area under the receiver operating characteristic curve (AUC). The AUC was the probability that the test ranked a randomly chosen stored profile as more likely to have been stored than a randomly chosen held-out profile; 0.5 indicated chance discrimination and 1.0 indicated perfect discrimination. The mean AUC was 0.97, and nine splits reached 1.0. We next asked whether this signal came from the numerical sampling procedure. A Metropolis-adjusted procedure left the mean AUC nearly unchanged at 0.96. Lowering the inverse temperature over a 40-fold range increased the median distance from synthetic profiles to their nearest stored profiles, but cross-visit covariance fidelity declined. Drawing directly from the target distribution, without a Markov chain, produced a mean AUC of 0.98 (Supplementary Table S16, Fig. S9). Thus, the membership signal came from the target distribution in this small-cohort setting, not from the Markov-chain implementation. The test could reveal whether an already-known de-identified profile had been stored, but it could not identify a person or reconstruct an unknown record.

### 2.6 Conditional Generation of Rare Subgroups

The previous analyses used unconditioned SA, generating from the full cohort. A key clinical application, however, is generating patients from specific subpopulations that are too small to study independently. We therefore tested whether SA could amplify rare clinical subpopulations while preserving their condition-specific signatures. We defined three overlapping subgroups by pregnancy outcome and comorbidity: Uncomplicated (n=18), PCOS (n=3, cross-cutting; 1 patient also in the PE group), and Developed PE $\left(n=5\right)$. The PCOS and Developed PE groups were too small for any independent statistical analysis. We used SA’s multiplicity-weighted sampling to generate condition-specific cohorts of 100 patients each by upweighting attention on the relevant stored patterns.

We compared condition-specific feature means between real and SA-generated patients across eight coagulation features that exhibited between-condition variation (Fig. 5). The generated cohorts preserved the condition-specific patterns. Patients with PCOS showed elevated Factor VIII and vWF relative to Healthy patients in both real and synthetic data, PE patients showed elevated $\alpha_{2}$-AP and Factor IX, and the between-condition rank ordering was maintained across all eight features. We then performed a descriptive bootstrap Mann–Whitney diagnostic. For each feature and condition, we subsampled the synthetic cohort to match the real sample size $\left(n_{\text{real}}\right)$, applied a Mann–Whitney U test, and repeated 1,000 times. We reported the fraction of replicates where *p* > 0.05, meaning that this test did not detect a difference in that replicate. This power-limited fraction was descriptive and did not establish equivalence. Across all 24 feature–condition pairs, 20 (83%) achieved *p* > 0.05 in ≥90% of replicates, with a median no-difference-detected fraction of 98.6% (full per-pair results in Table S10). The four pairs that fell below 90% were high-variance features in the smallest subgroups (FIX and plasminogen in PCOS; FIX and plasminogen in Developed PE), where the real data exhibited large inter-patient variability. No multiple-testing correction was applied because this diagnostic was not used to make simultaneous inferential claims; the per-pair values and the 90% threshold were descriptive reporting summaries. The PCA projections of the conditioned cohorts are provided in Figs. S3–S4. These results showed that SA’s multiplicity-weighted interpolation can amplify rare subgroups in a way that preserves their distinguishing clinical features, useful for exploratory hypothesis generation and simulation-based workflow testing, but not for increasing the effective clinical sample size. The multivariate-normal model cannot do this: fitting a separate MVN to three PCOS patients across 216 features is mathematically impossible (rank 2 covariance), and post-hoc subsetting of unconditional MVN samples does not preserve subgroup-specific structure. Together, these comparisons showed that multiplicity weighting preserved the subgroup patterns represented in the source cohort.

Generating 100 conditioned profiles did not add independent subgroup information. The PCOS and Developed PE signals still came from three and five real patients, and their participation ratios were 4.6 and 7.7 (Table S2). Thus, repeated draws were concentrated around a few stored profiles. Treating them as independent observations would understate uncertainty. They instead provided denser simulations of the distributions suggested by those patients for exploratory modeling and workflow testing.

### 2.7 Mechanistic Consistency

The preceding validations assessed statistical properties of the synthetic data. We next tested whether factor combinations generated by stochastic attention produced biologically plausible outputs when passed through a separately specified mechanistic model. The model was blind to the generation process but was calibrated on the same cohort. We used the Hockin–Mann BZ2012 model [Hockin et al., 2002, Brummel-Ziedins et al., 2012], a system of 58 ordinary differential equations with 64 rate constants that simulates thrombin generation from patient-specific coagulation factor inputs. We calibrated 5 of the 64 rate constants (prothrombinase, intrinsic and extrinsic tenase, protein C activation, and meizothrombin conversion; Table S4) on Visit 1 real patients at the population level and held the remaining 59 at published literature values. We then ran the calibrated model on all 23 real patients and 100 synthetic patients under both TF-only and TF+TM conditions (738 total simulations, 0 failures). For each patient, we computed five ODE-predicted thrombin generation features (lagtime, peak, time-to-peak, maximum rate, and ETP) and compared them against the corresponding values from the patient record. In these comparisons (Fig. 6, top row), the horizontal axis is the TGA value from the patient record, which exists for both real and synthetic patients, while the vertical axis is the value computed by the BZ2012 model from that patient’s factor inputs. Systematic deviations from the y=x line reflect ODE model bias (e.g., lagtime is underpredicted at ≈0.77×), not a failure of the synthetic data; the key observation is that real and synthetic patients share the same pattern of model bias, occupying the same cloud with the same systematic offset.

We formalized this observation by computing the ODE-predicted/dataset ratio for each patient and comparing the resulting distributions between real and synthetic populations (Fig. 6, bottom row). These ratio distributions capture how the ODE model processes each patient’s factor levels. If SA had generated patients with biologically implausible factor combinations, the model would process them differently, producing a shifted or broadened ratio distribution. Instead, the distributions overlapped substantially, with cloud overlap (fraction of synthetic ratios within the 5th–95th percentile of real ratios) ranging from 0.83 for ETP to 0.92 for T.Peak under TF-only conditions, and two-sample Kolmogorov–Smirnov (KS) tests did not detect a difference between the real and synthetic ratio distributions at this sample size across all five TGA features (*D* = 0.081–0.123, all *p* > 0.30; full diagnostics in Table S11). Under TF+TM conditions, the model systematically overcorrected the protein C anticoagulant pathway, producing peak and maximum rate predictions approximately 0.5× measured values for both real and synthetic patients (Fig. S5, same layout as the main-text comparison). Cloud overlap remained high (0.88–0.92 across the five features), showing similar model behavior for real and synthetic profiles despite the imperfect calibration.

The model was calibrated only on Visit 1 real patients, but the per-visit median predicted-to-measured ratios under TF-only conditions remained close to 1.0 at Visits 2 and 3 (Fig. S6). This was consistent with rate constants that remained useful beyond the calibration visit. Spearman rank correlations between predicted and measured values were moderate for ETP (ρ≈0.6–0.8 for real, ρ≈0.6–0.65 for synthetic; Fig. S7) and weak for other features, consistent with a 5-parameter population-level calibration that was not designed to resolve inter-patient variability. The key finding was not patient-level prediction but population-level plausibility. Applied identically to both groups, the mechanistic model produced overlapping predicted-to-recorded ratio distributions for real and synthetic patients under both experimental conditions. Because the model was calibrated on this same cohort, this was a same-cohort consistency check rather than independent validation.

### 2.8 Downstream Utility: Mechanistic Model Calibration

Having established same-cohort mechanistic consistency, we tested whether synthetic profiles could support mechanistic-model calibration. We fit the five BZ2012 rate constants under TF-only conditions to either 100 synthetic V1 profiles or 23 real V1 profiles, using the same bounded Nelder–Mead optimization and 11 random restarts (Table S4). We evaluated both calibrations on held-out real V2 and V3 TGA measurements. Median relative errors for the synthetic calibration were 2–10% lower across the five features, with an overall synthetic-to-real error ratio of 0.94 (Fig. 7; Table S12). The parameter estimates differed, but the two calibrations produced highly correlated predictions. Because the synthetic profiles came from the real cohort, this was not an independent validation. It tested whether the representation retained enough structure for calibration. The modest reduction in prediction error did not show that synthetic data were superior. It may instead have reflected a more stable calibration objective produced by sampling the represented distribution more densely. Together with the subgroup and factor-level analyses, this result supported exploratory modeling and hypothesis generation. Confirming those hypotheses and establishing clinical utility would require additional real patients or independent experimental data.

## 3 Discussion

This study asked whether a geometry-based method could generate synthetic profiles from a very small longitudinal cohort while preserving statistical and modeled biological relationships. Median feature MRE was 1.2%, which the MVN baseline also achieved. Stochastic attention also retained cross-visit covariance, whereas MVN introduced variance in 194 null dimensions. Conditioned generation preserved the subgroup patterns represented by three PCOS and five Developed PE patients. Finally, a separately specified, generator-blind coagulation model calibrated on the same cohort placed real and synthetic profiles in the same factor-to-thrombin-generation envelope. Cloud overlap was 83–92%, and the Kolmogorov–Smirnov tests did not detect differences under these conditions (*p* > 0.35 for all five TGA features).

Dense simulations from very small longitudinal datasets could support maternal health research. Rare pregnancy complications such as PCOS and preeclampsia are difficult to study because assembling large, well-characterized longitudinal cohorts requires years of recruitment across multiple clinical sites. Our results support hypothesis generation, computational modeling, and workflow testing with dense simulations of these small cohorts. Related work has predicted TGA responses from coagulation factors and searched for factor changes that produce a target response [Ghetmiri et al., 2021]. Although we did not evaluate either task with profiles generated by stochastic attention, these applications offer a clear direction for extending the present work.

Understanding why stochastic attention worked in this setting required examining the role of the PCA representation as well as the generator. When all methods used the same 18-dimensional subspace, the fitted two-component mixture and copula achieved marginal errors and mechanistic overlaps similar to those of stochastic attention. These results were therefore not unique to stochastic attention, although the comparison did not quantify the contribution of PCA itself. Empirical correlation error and novelty differed across generators. Stochastic attention used the patient profiles in the PCA subspace as its memory matrix and avoided estimating a full 216-dimensional covariance from 23 patients. Its sampler still required a choice of inverse temperature. A natural concern is whether the $\beta^{*}$ selection procedure, originally developed for protein sequence generation [Varner, 2026b], transfers to clinical coagulation data. The entropy transition rule selected the point of greatest downward curvature in attention entropy, a geometric property of K patterns in d dimensions rather than a property of what the features represented. For this dataset it gave $\beta^{*}\approx 2.94$, with the random-pattern scale $\sqrt{d}\approx 4.24$ serving as a theoretical reference. A sensitivity analysis showed gradual changes in generation quality across $\beta /\beta^{*}\in [0.1,3.0]$ (Table S1). Similarly, varying the PCA variance retention threshold from 85% to 99% ($d_{\text{PCA}}=13$ to 21) had minimal impact on generation quality. Median MRE remained between 1.2% and 1.8% across all thresholds (Table S7), so the 95% threshold was not a critical design choice. Together, these sensitivity analyses showed that performance was not tied to a narrow choice of inverse temperature or PCA threshold within the ranges tested.

Conditional generation raised a different question: how much diversity remained after the selected subgroup was upweighted? Achieving 80% of finite-mixture component probability on the PCOS subgroup required ρ≈26.7, reducing the participation ratio to $K_{\text{eff}}\approx 4.6$ (multiplicity parameters for all three subgroups in Table S2), which indicated that mixture-component probability was concentrated around a small number of stored profiles across repeated draws. The subgroup-specific signal still rested on only three real PCOS profiles, and the lower-weight background profiles pulled generated means toward the population average; $K_{\text{eff}}$ did not represent an independent patient count. Magnitudes were drawn from all 23 empirical PCA-space norms, so conditioning acted through the weighted directions rather than a subgroup-specific magnitude distribution. Thus, weighting concentrated sampling toward the subgroup directions while retaining some influence from the full cohort. Related studies have applied the same geometry-based operating-point rule and multiplicity weighting to protein sequence generation and conditional steering [Varner, 2026b,a, Alswaidan and Varner, 2026].

Among longitudinal clinical generators, VAMBN-MT provides a useful comparison in a larger-data setting [Kühnel et al., 2024]. It combines expert-defined modules, Bayesian-network constraints, and LSTM-augmented variational autoencoders. The authors evaluated it on the DONALD nutritional cohort and the Alzheimer’s Disease Neuroimaging Initiative. DONALD included 1,312 participants and 33 longitudinal variables, compared with 23 patients and 216 features here. Its best variant had cross-visit correlation error near 0.70, and weaker time trends were not reproduced at the tested sample sizes. The methods address different regimes. The VAMBN-MT model uses a trained modular architecture for moderate cohorts. Stochastic attention uses PCA geometry when n<p, applies a generic generator-blind mechanistic check instead of domain-specific rules, and uses multiplicity weights for conditional generation. This was a regime comparison, not a head-to-head test.

The mechanistic analysis addressed a different validation question: whether a model of the underlying biology processed real and synthetic profiles similarly. We processed both groups with the same separately specified model, which was blind to the generator but calibrated on the real cohort. A model calibrated on synthetic profiles also predicted held-out real TGA measurements as well as one calibrated on real profiles (error ratio 0.94, with 11 random restarts). The same comparison can be used wherever a validated mechanistic model exists, including pharmacokinetic [Mould and Upton, 2013], physiological [Chase et al., 2011], tumor-growth [Ribba et al., 2012], and metabolic [Yizhak et al., 2015, Shan et al., 2018] models. The relevant test is whether the model places real and synthetic profiles in the same predicted envelope.

The subgroup analysis focused on average differences between groups, but it did not show whether the conditioned cohorts preserved clinically important extremes. In the descriptive bootstrap Mann–Whitney diagnostic, the test detected no difference in fewer than 90% of replicates for four of the 24 feature–condition pairs: FIX and plasminogen in the PCOS and Developed PE groups. For these pairs, the relative differences between the real and synthetic subgroup means were 9–15%, and values varied widely among the real patients. This diagnostic had limited power and was not an equivalence test. The lower fractions could therefore reflect shifts in the means, a loss of extreme values, or simply the small samples. Truncating the PCA representation and drawing profile magnitudes from only 23 observed values could also make the generated extremes too narrow. If so, a synthetic cohort could not show how often severe states occur or how severe they become. Profiles outside the real cohort’s convex hull did not show that the true extremes were reproduced, and the primary mechanistic cloud-overlap measure considered only the real 5th–95th percentile range. We therefore do not recommend using these cohorts to estimate extreme risk without additional real data. The small source cohort caused other limits. Hull membership had little resolution, and the conditioned cohorts still relied on only three PCOS or five Developed PE patients. The modest calibration improvement was consistent with drawing more profiles from the same patient-derived distribution, not with adding biological information. The generated profiles supported simulation and model testing, but they did not add clinical evidence beyond the source cohort.

The pipeline required every patient to have the same assays at the same aligned visits. Concatenating the visits preserved relationships across visits, but the pipeline could not directly use irregular visit times, missing assays or visits, or different numbers of visits. Imputation, padding, or a sequence-aware method could support these records, but each would add assumptions that require validation in a small cohort. PCA added another limit because it captured only linear patterns. In the S-curve benchmark, generated profiles moved farther from the known curve as curvature increased. At every nonzero curvature, stochastic attention remained closer to the curve on average than the Gaussian generator, whereas the Gaussian generator recovered covariance more accurately. Neither generator was best on both measures. A nonlinear representation could better follow curved or branching patterns, but it would add choices about fitting the model and converting the generated values back to patient measurements. Those choices are difficult to make from 23 patients. The linear representation was practical for this cohort, but the results did not show that clinical trajectories are generally linear. Cohort size also affects computational cost. The reported Langevin sampler required work proportional to TKd for each profile, where T was the number of Langevin steps, K was the number of stored profiles, and d was the dimension of each profile in the sampling space. The exact mixture sampler removed the T sequential updates: its component weights could be prepared once, after which each profile required a component draw, a d-dimensional Gaussian draw, and conversion back to the original variables. The simulation advantage over the sample-covariance Gaussian occurred mainly in the n<p settings, and we did not test cohorts larger than 23 patients. The results therefore did not establish performance on irregular, incomplete, or strongly nonlinear records or in larger clinical cohorts.

This study had two broad limitations: the absence of independent clinical data and the restricted scope of the validation analyses. First, the clinical analysis used one longitudinal cohort of 23 patients. The known-covariance simulations tested selected properties under controlled conditions, and related protein studies applied the sampling framework in another domain, but neither established that the clinical findings would generalize to a different patient population. We did not test the method in a second clinical cohort, which is needed to assess that generalizability. Second, the validation analyses answered narrower questions than full biological or clinical validity. The BZ2012 model was calibrated on the same cohort used for generation. Applying one fixed, generator-blind model to real and synthetic profiles therefore tested consistency with that model, not independent biological validity. The fitted rate constants also required a 50-fold reduction in intrinsic tenase $k_{\text{cat}}$ and a 15-fold increase in extrinsic tenase $k_{\text{cat}}$ relative to published values (Table S4), likely because the published measurements and the plasma-based assay used different experimental conditions. These fitted values should not be read as new biochemical estimates. The biological scope was also incomplete. The mechanistic test covered thrombin generation but not the fibrinolytic or viscoelastic features. The membership-inference analysis answered a separate, narrow question: whether an already-known de-identified profile had been stored in the memory matrix. It distinguished stored from held-out profiles, but it did not identify a participant or reconstruct an unknown record. Finally, we did not test clinical outcomes. Thus, the results did not establish biological fidelity across the full profile or clinical utility. An independently calibrated model, models covering the other feature classes, and prospective outcome validation could address these gaps. The results establish proof of concept, not generalizability.

## 4 Conclusion

Multiplicity-weighted Stochastic Attention generated longitudinal profiles from 23 patients with low marginal error, retained cross-visit covariance, and preserved the represented subgroup patterns. The profiles were also consistent with a coagulation ODE model calibrated on the same cohort. A model calibrated on synthetic profiles predicted held-out real TGA measurements as well as one calibrated on real profiles. Direct sampling from the same target distribution reproduced the Langevin sampler’s fidelity and membership-inference results. Together, these findings provide proof of concept for using synthetic profiles in the modeling tasks tested here with very small longitudinal cohorts.

## 5 Methods

### 5.1 Population Dataset

We analyzed a longitudinal dataset of coagulation, fibrinolysis, thrombin generation assay (TGA), and rotational thromboelastometry (ROTEM) measurements from women desiring a pregnancy. The study was approved by the Institutional Review Board at the University of Vermont. All participants gave written consent before enrollment. Blood collection, plasma processing, and assay protocols have been described previously [McLean et al., 2012, Hale et al., 2012, Psoinos et al., 2021, Bernstein et al., 2016]. Briefly, citrated platelet-poor plasma was collected without tourniquet use after supine rest at three clinical visits: Visit 1 (pre-pregnancy baseline in the follicular phase), Visit 2 (end of first trimester), and Visit 3 (mid-third trimester). Plasma aliquots were stored at −80°C for subsequent analysis. Measurements of secondary analytes were measured as follows: Stago clotting assays measured coagulation-factor activity, ELISA measured fibrinolysis proteins, and the CLIA-certified laboratory at the University of Vermont Medical Center measured hormone levels. We performed TGA using 5 pM relipidated tissue factor with fluorogenic substrate on a Synergy4 plate reader as described in McLean et al.[McLean et al., 2012]. Clot formation and fibrinolysis dynamics were assessed via viscoelastometry using the ROTEM Delta Instrument (Werfen). Thawed plasma was mixed with or without 4 nM tPA (with respect to plasma volume), recalcified with 15 mM calcium chloride, and incubated for 3 minutes at 37°C. The reaction was initiated by adding the plasma mixture to ROTEM cups containing tissue factor at a 17:1 ratio; the final concentration of TF in the reaction was 5 pM.

The dataset contained 153 total records across approximately 50 subjects. After removing columns with > 30% missing values and retaining only subjects with complete data across all three visits, we obtained K=23 complete longitudinal profiles across n=72 assay measurements per visit. The 72 assay measurements spanned five categories: (i) hormones (estradiol, progesterone), (ii) coagulation factors and inhibitors (Factors II, V, VII, VIII, IX, X, XI, XII; antithrombin (AT), protein C (PC), tissue factor pathway inhibitor (TFPI), von Willebrand factor (vWF), fibrinogen, etc.), (iii) fibrinolytic markers (plasminogen, plasminogen activator inhibitor-1 (PAI-1), plasminogen activator inhibitor-2 (PAI-2), $\alpha_{2}$-antiplasmin ($\alpha_{2}$-AP), thrombin-activatable fibrinolysis inhibitor (TAFI), tissue plasminogen activator (tPA), D-dimer equivalents), (iv) thrombin generation parameters under four initiator conditions (TF at 5 pM, TF+thrombomodulin (TM), No TF, No TF+anti-FXIa), and (v) ROTEM/viscoelastic parameters (clotting time (CT), maximum clot firmness (MCF), alpha angle, lysis onset time (LOT), maximum lysis (ML), lysis time (LT), area under the curve (AUC) under TF Only and TF+tPA conditions).

Demographics and clinical characteristics of the resulting cohort, including age at enrollment, prepregnancy BMI, parity, gestational ages at each visit, and the cross-tabulation of enrollment cohort against pregnancy outcome, are summarized in Table 1. The 23 patients were drawn from three enrollment conditions: Healthy nulliparous women (n=14), women with a personal history of preeclampsia from a previous pregnancy (Prior PE, n=6), and women with polycystic ovarian syndrome (PCOS, n=3; 2 individuals were nulliparous). For conditional generation, we defined three overlapping subgroups based on pregnancy outcome and comorbidity rather than enrollment condition: Uncomplicated (n=18, all patients whose study pregnancy did not result in preeclampsia), PCOS (n=3, a cross-cutting comorbidity; 1 patient also developed PE), and Developed PE (n=5, patients who developed preeclampsia during the study pregnancy, drawn from 2 healthy-enrolled, 2 prior-PE-enrolled, and 1 PCOS-enrolled participants).

### 5.2 Multiplicity-Weighted Stochastic Attention

We generated synthetic patients using a multiplicity-weighted variant of Stochastic Attention (SA) rooted in modern Hopfield network theory [Ramsauer et al., 2021]. We stored K memory patterns as columns of a matrix $\hat{X}\in {\mathbb{R}}^{d\times K}$ and defined a weighted Hopfield energy landscape:

(1) $$E_{r}(\xi )=\frac{1}{2}\|\xi {\|}^{2}-\frac{1}{\beta }\log\sum_{k=1}^{K}r_{k}\;\exp\;\left(\beta \;m_{k}^{⊤}\xi \right)$$

where ${\left\{m_{k}\right\}}_{k=1}^{K}$ are stored memory patterns, β is an inverse temperature parameter controlling the sharpness of retrieval, and ${\left\{r_{k}\right\}}_{k=1}^{K}$ are per-pattern multiplicity weights. When all $r_{k}=1$, this reduces to the standard modern Hopfield energy; when $r_{k}=\rho >1$ for a designated subset of patterns, the energy landscape is continuously deformed to favor that subset.

This energy defines a probability distribution that can be written exactly. Writing $\pi_{r}(\xi )\propto \exp\left[-\beta E_{r}(\xi )\right]$ and completing the square in each exponent gives the expression:

(2) $$\exp\left[-\frac{\beta }{2}\|\xi {\|}^{2}+\beta m_{k}^{⊤}\xi \right]=\exp\left(\frac{\beta }{2}{\left\|m_{k}\right\|}^{2}\right)\exp\left[-\frac{\beta }{2}{\left\|\xi -m_{k}\right\|}^{2}\right],$$

Substituting Eq. (2) into the probability density gives an exact finite isotropic Gaussian mixture:

(3) $$\pi_{r}(\xi )=\sum_{k=1}^{K}q_{k}N\left(m_{k},\;\beta^{-1}I\right),\;q_{k}\propto r_{k}\exp\left(\frac{\beta }{2}{\left\|m_{k}\right\|}^{2}\right).$$

Each stored patient is the center of one component. The common component variance is $\beta^{-1}$, and the multiplicity weights enter the mixture probabilities directly. The memories used here are normalized to unit length, so the exponential factor is shared by every component and $q_{k}=r_{k}/\sum_{j}r_{j}$ exactly. We could therefore sample this distribution directly: first choose k from q and then draw $\xi \sim N\left(m_{k},\beta^{-1}I\right)$. This procedure requires no Markov chain. The cohort analyzed in this work was generated with the Langevin implementation given next, and that is what the reported results describe. We added the direct sampler for the stochastic-attention target afterward as a check, using the same normalization, magnitude rescaling, and decoding steps (Table S16).

We sampled from this landscape using an Unadjusted Langevin Algorithm (ULA):

(4) $$\xi_{t+1}=(1-\alpha )\xi_{t}+\alpha \hat{X}\;\mathrm{softmax}\left(\beta {\hat{X}}^{⊤}\xi_{t}+\log r\right)+\sqrt{\frac{2\alpha }{\beta }}\varepsilon_{t}$$

where α is a step size, $\varepsilon_{t}∼N(0,I)$, and logr is the element-wise log of the multiplicity vector. The deterministic component drove $\xi_{t}$ toward a multiplicity-weighted combination of stored patterns, while the stochastic component added variation. The $\text{log}\;r_{k}$ bias in the softmax logits interpolated between unconditioned generation (ρ=1, all patterns equally weighted) and hard subset curation (ρ→∞, only designated patterns contribute). This is an inference-time operation that requires no change to the stored patterns.

The synthetic cohort analyzed in this work was produced by initializing each Langevin chain near a randomly selected stored pattern and retaining the final state after T=2000 steps. Because the starting point and chain length could affect the output, we compared four sampling procedures. These used the original initialization near a stored pattern, random initialization on the unit sphere, pooled samples from multiple chains after burn-in and thinning, and Metropolis-adjusted sampling. As a separate check, we also drew directly from the exact stochastic-attention mixture in Eq. (3), without using a Markov chain. The fidelity and membership-inference results were similar across all four procedures and the direct draws. This agreement indicated that the original implementation reproduced the evaluated behavior of the sampling distribution at $\beta^{*}$ (Supplementary Fig. S9).

The inverse temperature β affects Hopfield retrieval and direct sampling in different ways. In the Hopfield update, small β spreads attention across many stored profiles and tends toward their weighted average, whereas large β concentrates attention on individual profiles. When sampling directly from Eq. (3), the stored profile is chosen according to $q_{k}$. For the unit-normalized profiles used here, these probabilities are independent of β and are set by the multiplicity weights. Instead, β controls the spread around the selected profile through the covariance $\beta^{-1}I$. Small β therefore produces broader draws, whereas large β produces tighter draws. The attention-entropy transition used to define $\beta^{*}$ marks the shift from attention shared across profiles to attention concentrated on individual profiles. We selected $\beta^{*}$ from the transition in normalized attention entropy across a logarithmic sweep of β. Specifically, $\beta^{*}$ was the grid point where the entropy curve bent downward most strongly, calculated as the most negative finite-difference second derivative with respect to logβ (Supplementary Methods). This choice depended on the relative positions of the K profiles in d dimensions, not on what their features represented. For this dataset (K=23, $d_{\text{PCA}}=18$), it gave $\beta^{*}\approx 2.94$; the random-pattern prediction $\sqrt{d}\approx 4.24$ served as a theoretical reference. A sensitivity analysis across $\beta /\beta^{*}\in [0.1,3.0]$ showed that the selected value balanced novelty and fidelity (Table S1). For conditioned generation, we repeated the entropy calculation with the multiplicity bias included, giving a ρ-dependent $\beta^{*}(\rho )$.

We used the participation ratio to measure how strongly multiplicity weighting concentrated repeated draws around a few stored profiles. For unit-normalized memories, $q_{k}=r_{k}/\sum_{j}r_{j}$ and $K_{\text{eff}}=1/\sum_{k}q_{k}^{2}={\left(\sum_{k}r_{k}\right)}^{2}/\sum_{k}r_{k}^{2}$. Thus, $K_{\text{eff}}$ is the number of equally weighted components with the same concentration. Equal weights gave $K_{\text{eff}}=23$; concentrating weight on three profiles moved it toward three. This was not an independent clinical sample size. To assign a target fraction $f_{\text{target}}$ of component probability to a subgroup, we used $\rho =f_{\text{target}}K_{\text{bg}}/\left[K_{\text{des}}\left(1-f_{\text{target}}\right)\right]$, where $K_{\text{des}}$ and $K_{\text{bg}}$ are the subgroup and background profile counts.

### 5.3 Pipeline for Continuous Longitudinal Data

Applying SA to continuous longitudinal clinical data required several adaptations beyond the standard Hopfield sampling framework. We first concatenated each patient’s n=72 assay measurements across all three visits into a single vector of dimension $d_{\text{concat}}=3n=216$, so that each stored memory pattern encoded a complete longitudinal profile and generated synthetic patients would have internally consistent multi-visit trajectories. We then standardized each feature to zero mean and unit variance and applied PCA retaining 95% of the variance, reducing dimensionality from 216 to $d_{\text{PCA}}=18$. This compression served two purposes: it removed collinearity among the 216 features and yielded a memory-to-dimension ratio of $K/d_{\text{PCA}}\approx 1.28$, placing SA in a favorable operating regime where the number of stored patterns exceeded the dimensionality of the representation.

Standard SA operates on unit-norm patterns, which is appropriate for discrete data (e.g., one-hot protein sequences) but destroys the anisotropic variance structure of continuous clinical measurements, collapsing dispersion across all principal components to a unit sphere. We addressed this with a direction-magnitude decomposition. Before normalization, we recorded each pattern’s PCA-space norm $s_{k}=\left\|m_{k}\right\|$; we then ran SA on unit-norm patterns to obtain a directional sample $\hat{\xi }$, drew a magnitude s from the empirical distribution of $\left\{s_{k}\right\}$, and reconstructed the rescaled sample as $\xi_{\text{rescaled}}=s\cdot \hat{\xi }$. This preserved the directional structure learned by the Hopfield energy while restoring the natural scale of variation. The rescaled PCA vector was mapped back to the original feature space via inverse PCA and destandardization, then split into three 72-dimensional visit records.

To generate condition-specific cohorts (e.g., PCOS-only), we set the multiplicity $r_{k}=\rho$ for patterns belonging to the target subgroup and $r_{k}=1$ for all others, and sampled using the weighted Langevin update (Eq. 4). The multiplicity ρ was computed to place a target fraction $f_{\text{target}}=0.80$ of the mixture-component probability on the designated subset, while retaining 20% probability on background patterns, via $\rho =f_{\text{target}}\cdot K_{bg}/\left(K_{\text{des}}\cdot \left(1-f_{\text{target}}\right)\right)$. Multiplicity parameters for all three subgroups are listed in Table S2. For the PCOS subgroup $\left(K_{\text{des}}=3\right)$, this required ρ≈26.7, reducing the participation ratio to $K_{\text{eff}}\approx 4.6$. For both unconditioned and conditioned generation, magnitudes were drawn from the empirical distribution of all 23 PCA-space norms. Subgroup weighting affected directional sampling, not the distribution of magnitudes.

The complete generation pipeline is summarized in Algorithm 1. The full set of SA hyperparameters used for all experiments is listed in Table S3, with key operating values α=0.01, T=2,000 Langevin iterations per sample, and random seed 42 for reproducibility. A control experiment comparing ULA with the Metropolis-Adjusted Langevin Algorithm (MALA) showed that the discretization bias is negligible at this step size. Across 10 chains of 5,000 iterations the MALA acceptance rate was 99.9 ± 0.03%, and mean post-burn-in energies and effective sample sizes were indistinguishable between the two samplers (Supplementary Table S8). The pipeline was implemented in Julia (v1.12) using the packages listed in the code repository. Generating 100 synthetic patients required approximately 0.1 seconds on a standard laptop (Apple M-series), comparable to MVN sampling (≈0.15 s), because SA operates in the 18-dimensional PCA space rather than the full 216-dimensional feature space. Each synthetic patient was generated by an independent Langevin chain (no shared state between patients); generation quality was stable across T∈[500,4000] iterations (median MRE 1.0–1.6%), indicating that T=2,000 was sufficient for convergence.

**Algorithm 1:** Multiplicity-Weighted SA for Longitudinal Patient Generation

**Require:** Patient data $\left\{x_{k}^{\left(v\right)}\right\}$ for k=1,…,K patients, v=1,2,3 visits		
**Require:** Multiplicity vector r (all ones for unconditioned; $r_{k}=\rho$ for designated subset)		
**Ensure:** N synthetic longitudinal patient profiles		
1:	Concatenate: ${\text{x}}_{k}\leftarrow \left[{\text{x}}_{k}^{\left(1\right)};{\text{x}}_{k}^{\left(2\right)};{\text{x}}_{k}^{\left(3\right)}\right]\in {\mathbb{R}}^{216}$	
2:	Standardize: $z_{k}\leftarrow \left(x_{k}-\mu \right)⊘\sigma$	
3:	PCA: $m_{k}\leftarrow W^{⊤}z_{k}\in {\mathbb{R}}^{18}$	▷ 95% variance retained
4:	Record norms: $s_{k}\leftarrow \left\|m_{k}\right\|$; normalize ${\hat{m}}_{k}\leftarrow m_{k}/s_{k}$	
5:	Select $\beta^{*}$ where the attention-entropy curve bends downward most strongly, including logr when conditioned	
6:	**for** i=1,…,N **do**	
7:	Select anchor $k_{0}$ from all patterns, or from the designated subset when conditioned	
8:	${\hat{\xi }}_{0}\leftarrow {\hat{m}}_{k_{0}}+0.01\varepsilon_{0}$; normalize ${\hat{\xi }}_{0}$	
9:	**for** t=0,…,T−1 **do**	▷ Langevin dynamics
10:	${\hat{\xi }}_{t+1}\leftarrow (1-\alpha ){\hat{\xi }}_{t}+\alpha \hat{M}\;\text{softmax}\left(\beta^{*}{\hat{M}}^{⊤}{\hat{\xi }}_{t}+\log r\right)+\sqrt{2\alpha /\beta^{*}}\varepsilon_{t}$	
11:	**end for**	
12:	Draw magnitude: $s∼{\left\{s_{k}\right\}}_{k=1}^{K}$	
13:	Rescale: $m_{\text{synth}}\leftarrow s\cdot {\hat{\xi }}_{T}/\left\|{\hat{\xi }}_{T}\right\|$	
14:	Inverse PCA + destandardize: ${\text{x}}_{\text{synth}}\leftarrow \sigma ⊙\left(Wm_{\text{synth}}+\bar{z}\right)+\mu$	
15:	Split into visit records: $x_{\text{synth}}^{\left(1\right)}$, $x_{\text{synth}}^{\left(2\right)}$, $x_{\text{synth}}^{\left(3\right)}$	
16:	**end for**	

### 5.4 Baseline and Validation Framework

As a baseline, we generated synthetic patients by fitting a single multivariate normal (MVN) distribution to the same K=23 concatenated 216-dimensional patient profiles. Both methods operated on the same concatenated representation. Each patient’s three visits were joined into one vector before generation, so that both methods had the opportunity to capture cross-visit dependencies. For SA, this structure was preserved through the Hopfield energy landscape operating on 18-dimensional PCA projections of the concatenated vectors. For MVN, the 216 × 216 sample covariance matrix had rank at most 22 and was regularized using Ledoit–Wolf shrinkage [Ledoit and Wolf, 2004], which inflated eigenvalues in the 194 null dimensions. To examine the role of the shared PCA representation, we also evaluated a fitted two-component diagonal-covariance Gaussian mixture, a kernel-density estimator, *k*-nearest-neighbor interpolation, and a Gaussian copula in the same 18-dimensional PCA space. Their implementations and shared evaluation pipeline are described in Supplementary Methods (PCA-space ablation). We then evaluated both SA and MVN synthetic patients through four progressively stringent validation levels. **Level 1 (Marginal Plausibility)** tested whether individual feature distributions matched, using per-feature means, standard deviations, and known physiological relationships. **Level 2 (Joint Structure)** tested whether the cross-visit covariance was preserved, by comparing full 216 × 216 correlation matrices, eigenvalue spectra, and PCA projections between real, SA, and MVN populations. **Level 3 (Conditional Structure)** tested whether rare subgroups could be amplified while preserving condition-specific signatures, by generating cohorts of 100 patients each from the Uncomplicated (n=18), PCOS $\left(n=3\right)$, and Developed PE $\left(n=5\right)$ subgroups using multiplicity-weighted sampling. **Level 4 (Mechanistic Consistency)** tested whether synthetic patients produced biologically plausible outputs under a separately specified, generator-blind mechanistic model calibrated on the same real cohort. We used the Hockin–Mann BZ2012 coagulation model [Hockin et al., 2002, Brummel-Ziedins et al., 2012], a system of 58 ODEs with 64 rate constants, in which 5 rate constants were calibrated on Visit 1 real patients at the population level and the remaining 59 were held at literature values. We ran the model on all real and synthetic patients under two conditions (TF-only and TF+TM) and compared predicted-versus-measured thrombin generation features. The primary metric was cloud overlap: the fraction of synthetic predicted/measured ratios falling within the 5th–95th percentile range of real ratios.

For the membership-inference analysis, we drew 40 random partitions of the 23 real profiles. For each partition, we repeated the preprocessing using 15 profiles, constructed the memory matrix, and generated 100 synthetic profiles; the other 8 real profiles were held out. We assigned each real profile a membership score equal to the negative Euclidean distance to its nearest synthetic profile in standardized 216-dimensional space, so a higher score indicated stronger evidence that the profile had been stored. A receiver operating characteristic curve compared the fraction of stored profiles correctly identified with the fraction of held-out profiles incorrectly identified as the score threshold changed. We calculated the area under this curve using the equivalent Mann–Whitney rank statistic, with ties contributing one half, and reported the mean across the 40 partitions. An AUC of 0.5 indicated chance discrimination, whereas 1.0 indicated perfect discrimination.

### 5.5 Robustness Tests

Results from one clinical cohort could not show how stochastic-attention generation behaved as training-set size, dimension, and nonlinear structure changed. We therefore used three tests. The covariance test compared stochastic attention with a sample-covariance Gaussian generator on low-rank Gaussian populations with known covariance. We generated one training cohort for every tested combination from the factor model $X=ZL^{⊤}+\epsilon$, where $Z\in {\mathbb{R}}^{n\times r}$ and $L\in {\mathbb{R}}^{p\times r}$ had independent standard-normal entries and ϵ had independent Gaussian entries with variance 0.1. This gave the known population covariance $\Sigma_{\text{pop}}=LL^{⊤}+0.1I$. We tested every combination of n∈{8,15,23,40,80}, p∈{30,120,216}, and r∈{3,5,10}. For each training cohort, we standardized the features, retained enough principal components to explain 95% of the variance, constructed the stochastic-attention memory matrix from the resulting scores, and selected β separately using the same attention-entropy criterion as in the clinical analysis. Stochastic attention generated 100 profiles using ULA with α=0.01, T=2,000, and empirical magnitude rescaling. The Gaussian generator used the training mean and sample covariance. For numerical sampling, diagonal jitter began at $10^{-8}I$ and increased tenfold as needed through $10^{-2}I$; if Cholesky sampling still failed, we sampled in the nonnegative eigenspace of the sample covariance. We applied no covariance shrinkage. The Gaussian generator also produced 100 profiles. Covariance error was ${\left\|{\hat{\Sigma }}_{\text{gen}}-\Sigma_{\text{pop}}\right\|}_{F}/{\left\|\Sigma_{\text{pop}}\right\|}_{F}$. Figure 4A reports Gaussian error divided by stochastic-attention error, averaged across the three latent ranks. A ratio above one favored stochastic attention.

The remaining tests asked how stochastic attention changed with less clinical information and how it compared with the Gaussian generator on a nonlinear population. For the sample-size test, we drew ten subsets without replacement from the 23 complete patients at each n∈{20,15,10,8}. For every subset, we repeated the standardization, PCA, and β selection, constructed a new stochastic-attention memory matrix, generated 100 profiles, and evaluated the generated cohort against the same 23-patient reference. The outcomes were relative cross-visit correlation error, pooled median marginal relative error, and TF-only mechanistic cloud overlap. Figure 4B shows the mean and standard deviation of cross-visit error across the ten subsets. For the nonlinearity test, we drew t uniformly from [−1, 1] and defined an S-curve by $x_{1}=t$ and $x_{2}=c\sin(\pi t)$. Viewed in the $\left(x_{1},x_{2}\right)$ plane, this one-dimensional path resembled the letter S because it had two opposing bends. Setting c=0 produced a straight line, and increasing c made the bends stronger. Each simulated profile was a noisy point near this path. For each replicate, we used a new random transformation to spread the two coordinates across p=30 or 120 simulated variables while preserving the curve’s geometry. We then added Gaussian noise with standard deviation 0.05 in every direction. We used n=23 and curvature c∈{0,0.25,0.5,1,1.5,2}, with five replicates per setting. From every training cohort, we constructed a stochastic-attention memory matrix and estimated the same sample-covariance Gaussian comparator; each generator produced 100 profiles. We projected generated profiles onto the known two-dimensional embedding plane and measured their mean distance from the curve, thereby excluding noise orthogonal to that plane. We also measured relative covariance error against the covariance of an independent 50,000-profile population draw. For the reported curvature threshold, we averaged stochastic-attention curve distance across the five replicates and both dimensions at each curvature and identified the first nonzero value at which this mean was at least twice its value for the straight-line population. All simulation cells used deterministic, cell-specific random-number streams derived from seed 42.

## Supplementary Material

Supplement 1

## Figures and Tables

**Figure 1:**
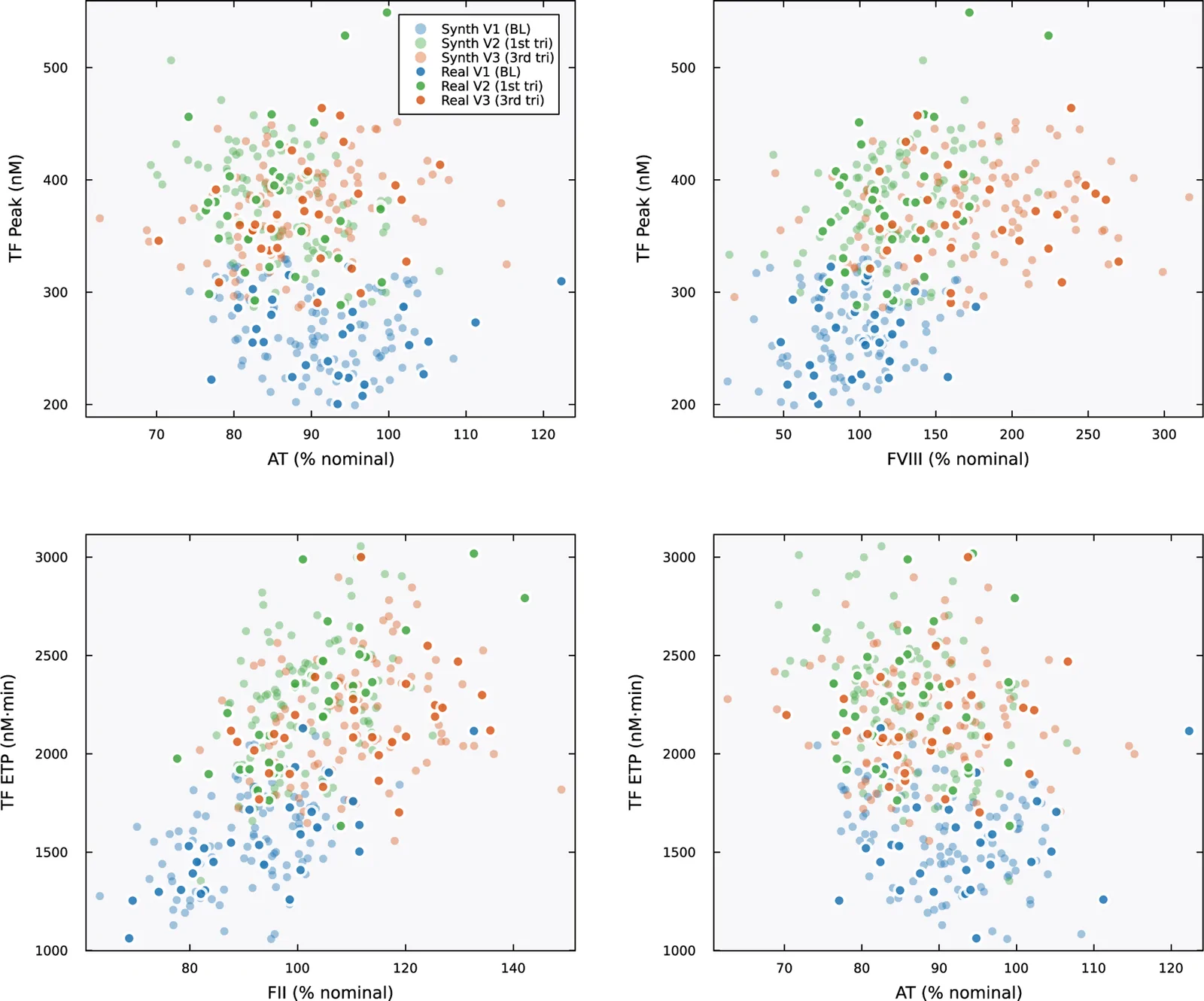
Physiological correlations. Scatter plots of coagulation factor levels versus thrombin generation parameters across all three visits. Filled markers are real patients; open markers are SA-generated synthetic patients. Colors encode visit: blue (V1/baseline), green (V2/first trimester), orange (V3/third trimester). Real and synthetic patients occupy the same joint regions across all four pairwise relationships. AT inversely correlates with TF-initiated peak thrombin (top left), FVIII positively correlates with peak (top right), and both FII (bottom left) and AT (bottom right) show the expected relationships with ETP. The visit-dependent shift in these relationships, reflecting the pregnancy-driven increase in procoagulant factors, is also preserved in the synthetic cohort.

**Figure 2:**
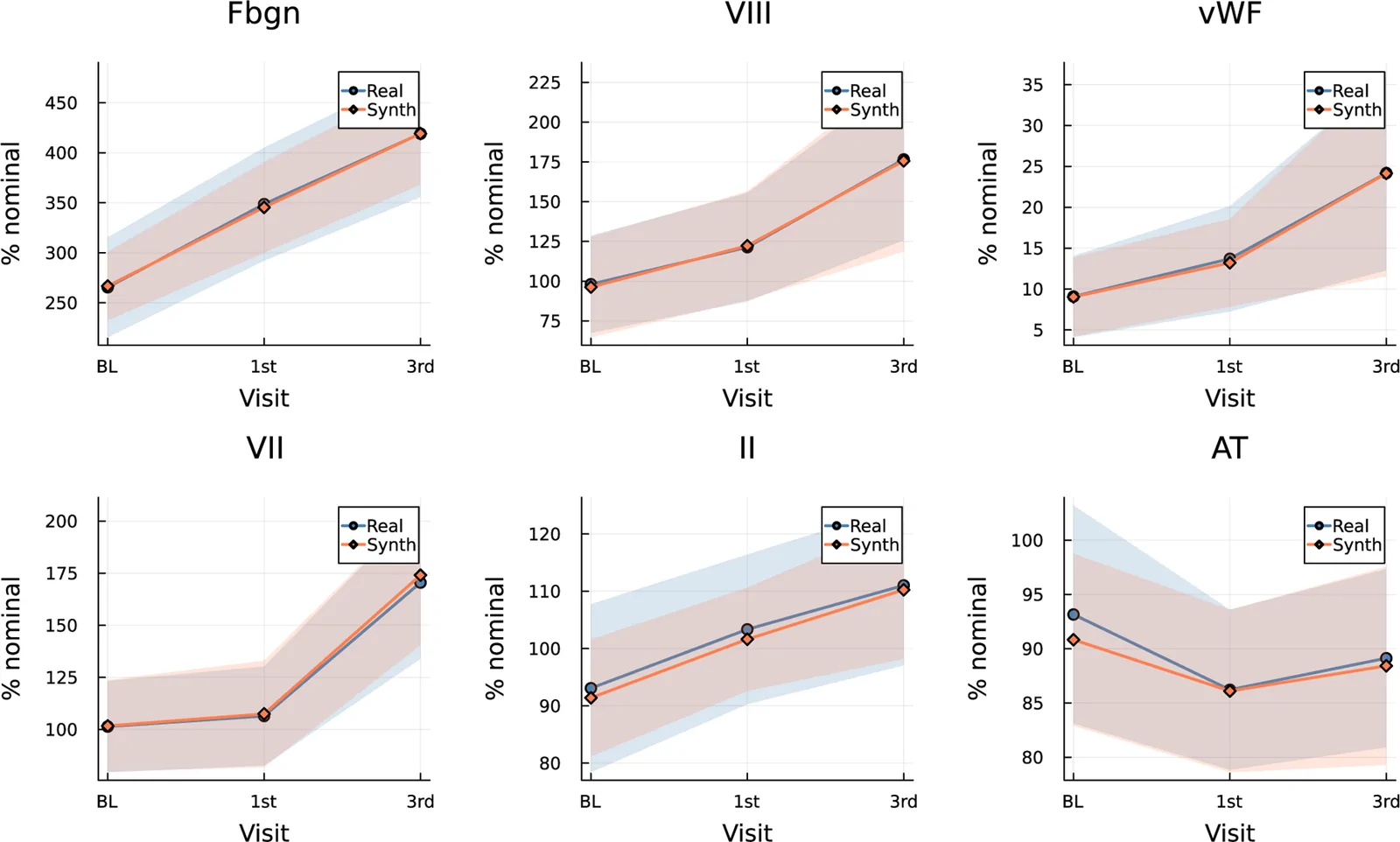
Pregnancy-related longitudinal trajectories. Mean ± standard deviation bands for six key coagulation features across visits (BL = baseline, 1st = first trimester, 3rd = third trimester) for real (blue) and SA-generated synthetic (orange) patients. The characteristic pregnancy-driven increases in fibrinogen, Factor VIII, and vWF are captured, as are the stable-to-declining patterns in Factor II and AT.

**Figure 3:**
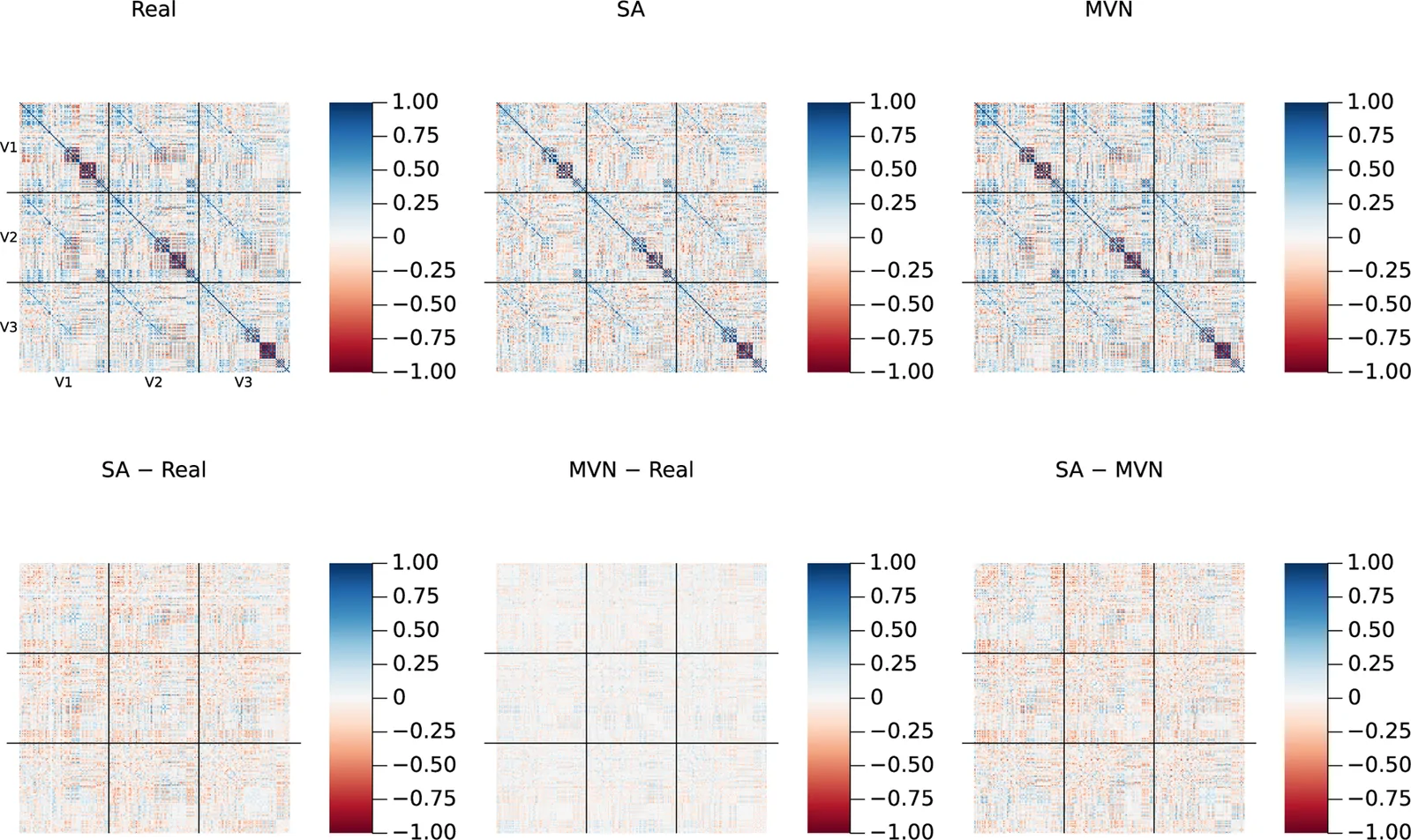
Cross-visit correlation structure. Top row: full 216 × 216 Pearson correlation matrices for Real (K=23, left), SA (N=100, center), and MVN (N=100, right) populations. Each matrix is organized as a 3 × 3 grid of 72 × 72 blocks (delineated by black lines), where the diagonal blocks capture within-visit feature correlations (V1–V1, V2–V2, V3–V3) and the off-diagonal blocks capture cross-visit dependencies (e.g., V1–V3 correlations between baseline and third-trimester Factor VIII). Stochastic attention preserves both the within-visit and cross-visit block structure. Bottom row: pairwise residual matrices (element-wise subtraction), all plotted on the same ±1 color scale as the top row. The SA–Real residual shows small, unstructured deviations. The MVN–Real residual is smoother, particularly in the off-diagonal blocks, reflecting the Ledoit–Wolf regularization that systematically shrinks cross-visit correlations toward zero. The SA–MVN residual shows the largest differences in these off-diagonal blocks, where SA retains longitudinal dependencies that MVN’s rank-deficient covariance estimate cannot represent. A version with amplified residual color scale (±0.5) and Frobenius norms is provided in Fig. S1.

**Figure 4:**
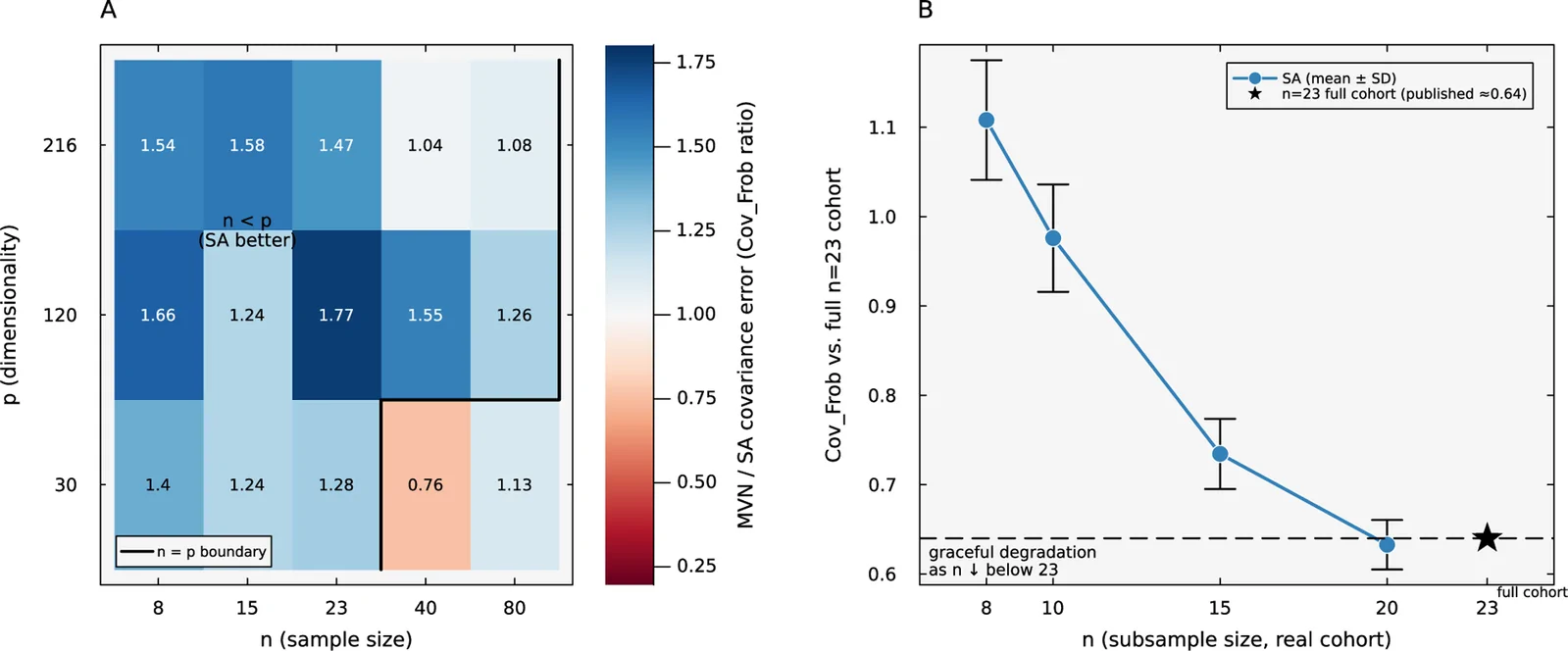
Population recovery and clinical-cohort subsampling. (A) For each low-rank Gaussian setting, the stochastic-attention memory matrix was constructed and a sample-covariance Gaussian generator was estimated from the same simulated training cohort; both generators produced 100 profiles. Color represented the ratio of their relative Frobenius errors against the known population covariance (Gaussian error divided by stochastic-attention error), averaged over latent ranks 3, 5, and 10. Values above one (blue) indicated lower error for stochastic attention. Because the target covariance was fixed before the training cohort was drawn, this measured population recovery rather than reproduction of the training data. (B) A stochastic-attention memory matrix was constructed from each of ten random subsets of the clinical cohort at n=20,15,10, and 8, and each matrix generated 100 profiles. Points and bars represented the mean and standard deviation of the relative cross-visit correlation error against the same full 23-patient reference; the star represented generation using all 23 patients.

**Figure 5:**
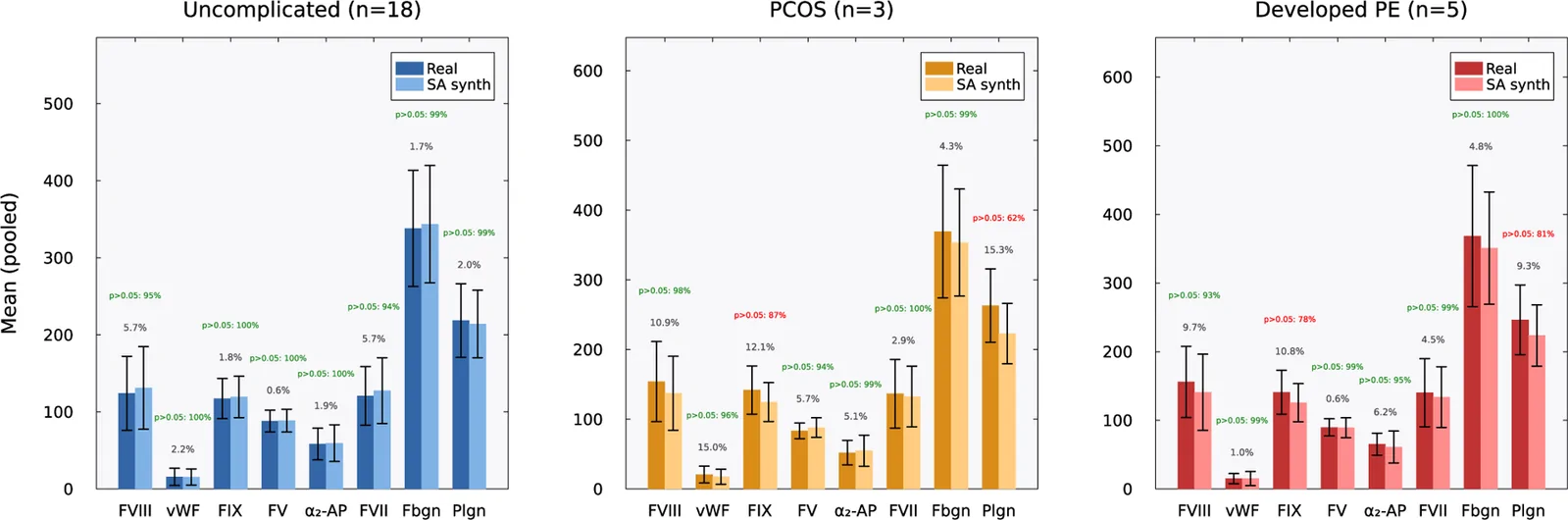
Condition-specific feature preservation. Grouped bar charts comparing real (dark bars) and SA-generated synthetic (light bars) patient means (± SD error bars) for eight coagulation features, shown separately for each clinical subgroup: Uncomplicated (n=18, left, blue), PCOS (n=3, center, orange), and Developed PE (n=5, right, red). Each bar is the mean of that assay pooled across all three visits (V1–V3); factor activities (FVIII, vWF, FIX, FV, $\alpha_{2}$-AP, FVII, plasminogen) are expressed as % of normal pooled reference plasma, and fibrinogen (Fbgn) in mg/dL. Gray percentages indicate the mean relative error (MRE). Green annotations show a descriptive bootstrap Mann–Whitney diagnostic: the fraction of 1,000 bootstrap replicates (synthetic subsampled to $n_{\text{real}}$) in which *p* > 0.05, meaning that this test detected no difference at the available sample size. This is not an equivalence test. Features achieving ≥90% are shown in green; <90% in red. Across all 24 feature–condition pairs, 20 (83%) had no difference detected in ≥90% of replicates (median: 98.6%). The four pairs below this threshold were FIX and plasminogen in the PCOS and Developed PE subgroups, where small subgroup sizes and large inter-patient variability reduced test power.

**Figure 6:**
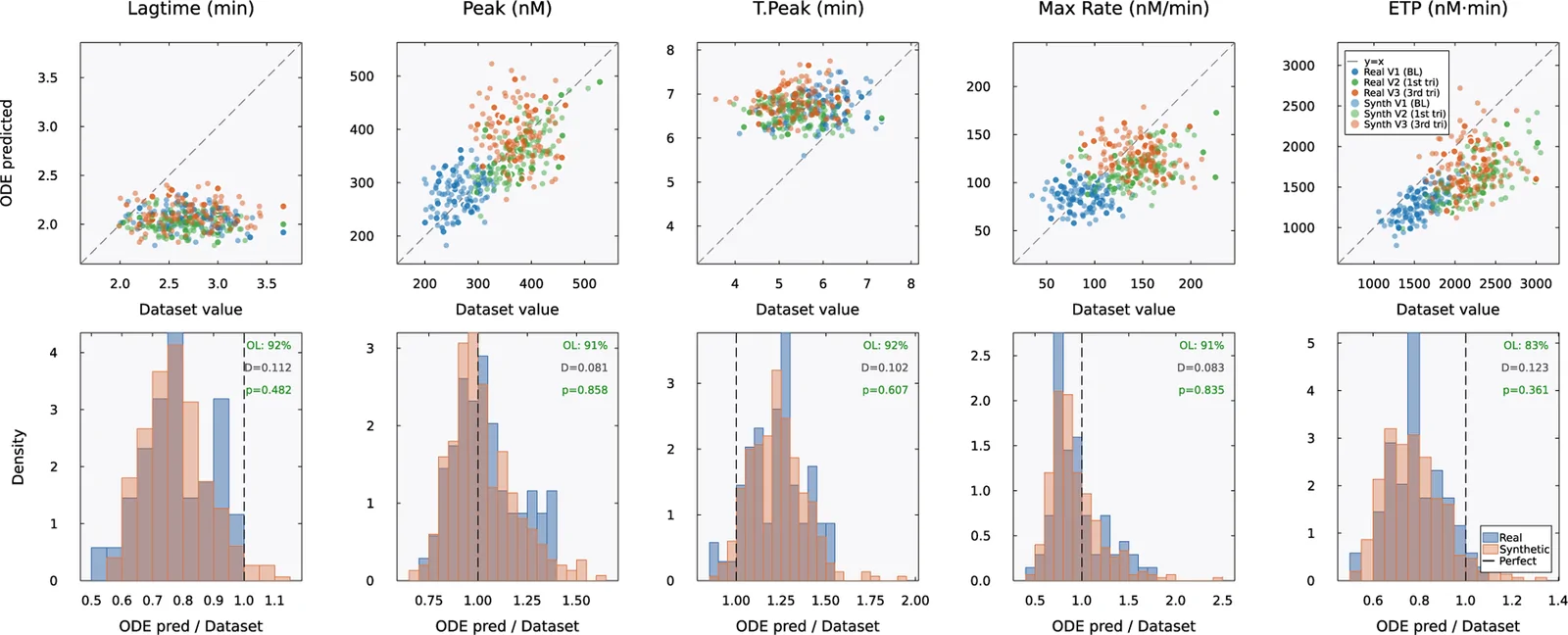
Mechanistic validation (TF-only). Top row: BZ2012 ODE-predicted TGA values (vertical axis) versus dataset TGA values (horizontal axis) for five thrombin generation parameters. The dataset values are the TGA measurements from each patient’s record, present for both real and synthetic patients. The ODE-predicted values are computed by running each patient’s coagulation factor levels through the 58-species BZ2012 model. The dashed line indicates where the ODE model would perfectly reproduce the dataset value (y=x); systematic deviations from this line (e.g., lagtime underprediction) reflect ODE model bias, not synthetic data failure. Filled markers are real patients, open markers are SA-generated synthetic patients, colored by visit: blue (V1/baseline, training set), green (V2/first trimester), orange (V3/third trimester). Real and synthetic patients occupy the same cloud with the same pattern of ODE model bias. Bottom row: ODE-predicted/dataset ratio distributions for real (blue) and synthetic (orange) patients. The distributions overlap substantially. Each panel is annotated with cloud overlap (OL), the two-sample Kolmogorov–Smirnov statistic (D), and *p*-value. All five features had *p* > 0.35; these tests did not detect a difference between real and synthetic ratio distributions.

**Figure 7:**
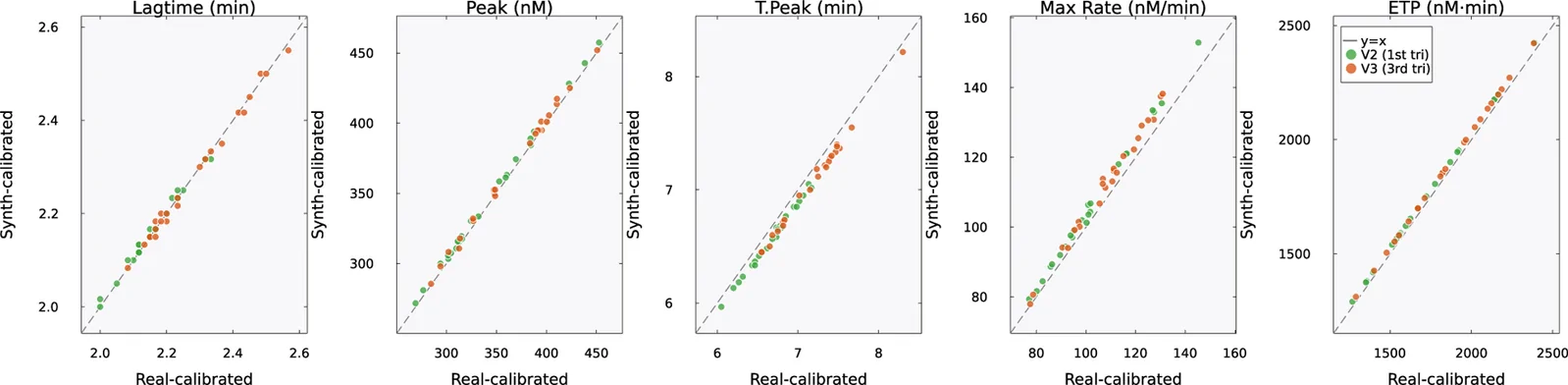
Downstream utility: synthetic and real calibrations on held-out real profiles. Each panel compares the BZ2012 ODE predictions for one TGA feature when the model is calibrated on real V1 patients (horizontal axis) versus synthetic V1 patients (vertical axis), evaluated on the same held-out real V2 and V3 patients. Points near the y=x line indicate similar predictions from the two calibrations. Green: V2 (first trimester); orange: V3 (third trimester). The synthcalibrated model had slightly lower median relative error on all five features (overall synthetic-to-real error ratio 0.94). Both calibrations used 11 random restarts; all starts converged.

**Table 1:** Demographics and clinical characteristics of the 23 patients with complete longitudinal data across all three visits. Values are reported as mean ± SD, median (range), or *n* (%) as appropriate.

Characteristic	Mean ± SD	Median (Range)	*n* (%)

Age at enrollment (yrs)	30.2 ± 5.1	29 (20–41)	
Race			
White			22 (96)
Not disclosed			1 (4)
Prepregnancy BMI	26.6 ± 5.3	25 (19.0–37.8)	
Parity		0 (0–2)	
Nulliparous			16 (70)
Parous			7 (30)
Enrollment condition			
Healthy nulliparous			14 (61)
PCOS			3 (13)
Prior PE			6 (26)
Cycle day at V1	9.2 ± 3.6	10 (3–14)	
Gestational age at V2 (days)	89.0 ± 5.5		
Gestational age at V3 (days)	217.0 ± 4.0		
*Enrollment cohort × pregnancy outcome*			
	Uncomplicated	Developed PE	Total

Healthy nulliparous	12	2	14
Prior PE	4	2	6
PCOS	2	1	3

Total	18	5	23

**Table 2:** Per-visit mean relative error (MRE) between real and SA-generated synthetic patients for representative coagulation features, computed as $\left|{\bar{x}}_{\text{synth}}-{\bar{x}}_{\text{real}}\right|/\left|{\bar{x}}_{\text{real}}\right|$.

Feature	Visit 1	Visit 2	Visit 3
Factor II (%)	0.018	0.017	0.007
Factor VIII (%)	0.018	0.008	0.005
AT (%)	0.025	0.001	0.008
Fibrinogen (mg/dL)	0.005	0.009	<0.001
TF Peak (nM)	0.025	0.002	0.017
TF ETP (nM·min)	0.013	0.008	0.023

## Data Availability

The code for synthetic patient generation, all validation scripts, and the generated synthetic datasets are available at https://github.com/varnerlab/SA-generation-legacy-dataset-paper . The real patient data were collected under approval from the University of Vermont Institutional Review Board; de-identified data are available upon reasonable request to the corresponding author.

## References

[R1] Toy Sarah. U.S. maternal mortality hits highest level since 1965. The Wall Street Journal, 2023. March 16, 2023.

[R2] Harris Emily. US maternal mortality continues to worsen. JAMA, 329(15):1248, 2023. doi: 10.1001/jama.2023.5254. 36988993

[R3] Chen Yingxi, Shiels Meredith S, Uribe-Leitz Tarsicio, Molina Rose L, Lawrence Wayne R, Freedman Neal D, and Abnet Christian C. Pregnancy-related deaths in the US, 2018–2022. JAMA Network Open, 8(4):e254325, 2025. doi: 10.1001/jamanetworkopen.2025.4325. PMC11983229 40202759

[R4] Mann Kenneth G. Thrombin generation in hemorrhage control and vascular occlusion. Circulation, 124(2):225–235, 2011. doi: 10.1161/CIRCULATIONAHA.110.952648. PMC3138077 21747067

[R5] Butenas Saulius, Orfeo Thomas, and Mann Kenneth G. Tissue factor in coagulation: Which? where? when? Arteriosclerosis, Thrombosis, and Vascular Biology, 29(12):1989–1996, 2009. doi: 10.1161/ATVBAHA.108.177402. PMC2783287 19592470

[R6] Hockin Mark F, Jones Kenneth C, Everse Stephen J, and Mann Kenneth G. A model for the stoichiometric regulation of blood coagulation. Journal of Biological Chemistry, 277(21):18322–18333, 2002. doi: 10.1074/jbc.M201173200. 11893748

[R7] Bravo Maria C, Orfeo Thomas, Mann Kenneth G, and Everse Stephen J. Modeling of human factor Va inactivation by activated protein C. BMC Systems Biology, 6:45, 2012. doi: 10.1186/1752-0509-6-45. PMC3403913 22607732

[R8] Hellgren Margareta. Hemostasis during normal pregnancy and puerperium. Seminars in Thrombosis and Hemostasis, 29(2):125–130, 2003. doi: 10.1055/s-2003-38897. 12709915

[R9] Grouzi E, Pouliakis A, Aktypi A, Pregnancy and thrombosis risk for women without a history of thrombotic events: a retrospective study of the real risks. Thrombosis Journal, 20(60), 2022. doi: 10.1186/s12959-022-00419-6. PMC9535874 36203153

[R10] Bremme Katarina A. Haemostatic changes in pregnancy. Best Practice & Research Clinical Haematology, 16(2):153–168, 2003. doi: 10.1016/S1521-6926(03)00021-5. 12763484

[R11] Azziz Ricardo, Woods Keslie S, Reyna Rosario, Key Thomas J, Knochenhauer Eric S, and Yildiz Bulent O. The prevalence and features of the polycystic ovary syndrome in an unselected population. Journal of Clinical Endocrinology & Metabolism, 89(6):2745–2749, 2004. doi: 10.1210/jc.2003-032046. 15181052

[R12] Palomba Stefano, Santagni Susanna, Falbo Angela, and La Sala Giovanni Battista. Complications and challenges associated with polycystic ovary syndrome: current perspectives. International Journal of Women’s Health, 7:745–763, 2015. doi: 10.2147/IJWH.S70314. PMC4527566 26261426

[R13] Abalos Edgardo, Cuesta Cristina, Carroli Guillermo, Qureshi Zahida, Widmer Mariana, Vogel Joshua P, and Souza João Paulo. Pre-eclampsia, eclampsia and adverse maternal and perinatal outcomes: a secondary analysis of the World Health Organization multicountry survey on maternal and newborn health. BJOG: An International Journal of Obstetrics & Gynaecology, 121:14–24, 2014. doi: 10.1111/1471-0528.12629. 24641531

[R14] Dusse Luci M, Rios Danyelle R A, Pinheiro Melina B, Cooper Alan J, and Lwaleed Bashir A. Pre-eclampsia: relationship between coagulation, fibrinolysis and inflammation. Clinica Chimica Acta, 412(1–2):17–21, 2011. doi: 10.1016/j.cca.2010.09.030. 20888809

[R15] Luan Deyan, Zai Michael, and Varner Jeffrey D. Computationally derived points of fragility of a human cascade are consistent with current therapeutic strategies. PLoS Computational Biology, 3(7):e142, 2007. doi: 10.1371/journal.pcbi.0030142. PMC1924874 17658944

[R16] Luan Deyan, Szlam Fania, Tanaka Kenichi A, Barie Philip S, and Varner Jeffrey D. Ensembles of uncertain mathematical models can identify network response to therapeutic interventions. Molecular BioSystems, 6(11):2272–2286, 2010. doi: 10.1039/b920693k. 20844798

[R17] Brummel-Ziedins Kathleen E, Orfeo Thomas, Callas Peter W, Gissel Matthew, Mann Kenneth G, and Bovill Edwin G. The prothrombotic phenotypes in familial protein c deficiency are differentiated by computational modeling of thrombin generation. PLoS ONE, 7(9):e44378, 2012. doi: 10.1371/journal.pone.0044378. PMC3440432 22984498

[R18] Hastie Trevor, Tibshirani Robert, and Friedman Jerome. The Elements of Statistical Learning: Data Mining, Inference, and Prediction. Springer, 2nd edition, 2009. doi: 10.1007/978-0-387-84858-7.

[R19] Gonzales Aldren, Guruswamy Guruprabha, and Smith Scott R. Synthetic data in health care: A narrative review. PLOS Digital Health, 2(1):e0000082, 2023. doi: 10.1371/journal.pdig.0000082. PMC9931305 36812604

[R20] Kühnel Lisa, Schneider Julian, Perrar Ines, Adams Tim, Moazemi Sobhan, Prasser Fabian, Nöthlings Ute, Fröhlich Holger, and Fluck Juliane. Synthetic data generation for a longitudinal cohort study – evaluation, method extension and reproduction of published data analysis results. Scientific Reports, 14:14412, 2024. doi: 10.1038/s41598-024-62102-2. PMC11193715 38909025

[R21] Ledoit Olivier and Wolf Michael. A well-conditioned estimator for large-dimensional covariance matrices. Journal of Multivariate Analysis, 88(2):365–411, 2004. doi: 10.1016/S0047-259X(03)00096-4.

[R22] Goodfellow Ian, Pouget-Abadie Jean, Mirza Mehdi, Xu Bing, Warde-Farley David, Ozair Sherjil, Courville Aaron, and Bengio Yoshua. Generative adversarial nets. In Advances in Neural Information Processing Systems, volume 27, pages 2672–2680, 2014.

[R23] Kingma Diederik P and Welling Max. Auto-encoding variational Bayes. In International Conference on Learning Representations (ICLR), 2014.

[R24] Xu Lei, Skoularidou Maria, Cuesta-Infante Alfredo, and Veeramachaneni Kalyan. Modeling tabular data using conditional GAN. In Advances in Neural Information Processing Systems, volume 32, pages 7335–7345, 2019.

[R25] De Mitri Omar, Wang Ruyu, and Huber Marco F. Generative adversarial networks with limited data: A survey and benchmarking. arXiv preprint arXiv:2504.05456, 2025. doi: 10.48550/arXiv.2504.05456.

[R26] Ramsauer Hubert, Bernhard Schäfl, Lehner Johannes, Seidl Philipp, Widrich Michael, Adler Thomas, Gruber Lukas, Holzleitner Markus, Milena Pavlović Geir Kjetil Sandve, Greiff Victor, Kreil David, Kopp Michael, Klambauer Gunter, Brandstetter Johannes, and Hochreiter Sepp. Hopfield networks is all you need. In International Conference on Learning Representations (ICLR), 2021.

[R27] Varner Jeffrey D. Conditioning protein generation via Hopfield pattern multiplicity. arXiv preprint arXiv:2603.20115, 2026a. doi: 10.48550/arXiv.2603.20115.

[R28] Bravo Maria, Orfeo Thomas, Bernstein Ira, Vadhin Sandra, Lakshmi Divya, and Varner Jeffrey D. BSTModelKit.jl: Building biochemical systems theory models of individualized coagulation dynamics during pregnancy. JuliaCon 2023, https://www.youtube.com/watch?v=IGtUqMLHBvQ , 2023. Conference proceedings talk.

[R29] Ghetmiri Damon E, Cohen Mitchell J, and Menezes Amor A. Personalized modulation of coagulation factors using a thrombin dynamics model to treat trauma-induced coagulopathy. npj Systems Biology and Applications, 7(1):44, 2021. doi: 10.1038/s41540-021-00202-9. PMC8651743 34876597

[R30] Varner Jeffrey D. Training-free generation of protein sequences from small family alignments via stochastic attention. arXiv preprint arXiv:2603.14717, 2026b. doi: 10.48550/arXiv.2603.14717.

[R31] Alswaidan Abdulrahman and Varner Jeffrey D. Stochastic attention via Langevin dynamics on the modern Hopfield energy. arXiv preprint arXiv:2603.06875, 2026. doi: 10.48550/arXiv.2603.06875.

[R32] Mould Diane R and Upton Richard N. Basic concepts in population modeling, simulation, and model-based drug development—part 2: introduction to pharmacokinetic modeling methods. CPT: Pharmacometrics & Systems Pharmacology, 2(4):e38, 2013. doi: 10.1038/psp.2013.14. PMC3636497 23887688

[R33] Chase J Geoffrey, Le Compte Aaron J, Preiser Jean-Charles, Shaw Geoffrey M, Penning Sophie, and Desaive Thomas. Physiological modeling, tight glycemic control, and the ICU clinician: what are models and how can they affect practice? Annals of Intensive Care, 1:11, 2011. doi: 10.1186/2110-5820-1-11. PMC3224460 21906337

[R34] Ribba Benjamin, Kaloshi Gentian, Peyre Mathieu, Ricard Damien, Calvez Vincent, Tod Michel, Cajavec-Bernard Branka, Idbaih Ahmed, Psimaras Dimitri, Dainese Luca, Pallud Johan, Cartalat-Carel Stéphanie, Delattre Jean-Yves, Honnorat Jérôme, Grenier Emmanuel, and Ducray François. A tumor growth inhibition model for low-grade glioma treated with chemotherapy or radiotherapy. Clinical Cancer Research, 18(18):5071–5080, 2012. doi: 10.1158/1078-0432.CCR-12-0084. 22761472

[R35] Yizhak Keren, Chaneton Barbara, Gottlieb Eyal, and Ruppin Eytan. Modeling cancer metabolism on a genome scale. Molecular Systems Biology, 11(6):817, 2015. doi: 10.15252/msb.20145307. PMC4501850 26130389

[R36] Shan Mengrou, Dai David, Vudem Arunodai, Varner Jeffrey D, and Stroock Abraham D. Multiscale computational study of the Warburg effect, reverse Warburg effect and glutamine addiction in solid tumors. PLoS Computational Biology, 14(12):e1006584, 2018. doi: 10.1371/journal.pcbi.1006584. PMC6285468 30532226

[R37] McLean Kelley C, Bernstein Ira M, and Brummel-Ziedins Kathleen. Tissue factor dependent thrombin generation across pregnancy. American Journal of Obstetrics and Gynecology, 207(2):135.e1–135.e6, 2012. doi: 10.1016/j.ajog.2012.05.027. PMC3661010 22840726

[R38] Hale Sarah A, Sobel Burton, Benvenuto Anna, Schonberg Adrienne, Badger Gary J, and Bernstein Ira M. Coagulation and fibrinolytic system protein profiles in women with normal pregnancies and pregnancies complicated by hypertension. Pregnancy Hypertension, 2(2):152–157, 2012. doi: 10.1016/j.preghy.2012.01.004. PMC3375860 22712057

[R39] Psoinos Rachel B C, Morris Erin A, McBride Carole A, and Bernstein Ira M. Association of pre-pregnancy subclinical insulin resistance with cardiac dysfunction in healthy nulliparous women. Pregnancy Hypertension, 26:11–16, 2021. doi: 10.1016/j.preghy.2021.07.246. PMC8616773 34392165

[R40] Bernstein Ira M, Hale Sarah A, and Badger Gary J. Differences in cardiovascular function comparing prior preeclamptics with nulliparous controls. Pregnancy Hypertension, 6(4):320–326, 2016. doi: 10.1016/j.preghy.2016.07.001. PMC5157840 27939476

