# Supplementary material for: Validated Synthetic Patient Generation for Small Longitudinal Cohorts: Coagulation Dynamics Across Pregnancy
Source: ArXiv. 2026 Aug 11:arXiv:2604.07557v2. Preprint. [Version 2] (PMC13640865)
Supplement: Supplementary file 1 [file NIHPP2604.07557v2-supplement-1.pdf]

Supplementary Information

Supplementary Methods

PCA-space ablation

We compared the canonical stochastic-attention cohort with four baseline generators operating on the same unnormalized 18-dimensional PCA coordinates. Each baseline generated 100 profiles with a separate random-number stream initialized using seed 42. The fitted two-component Gaussian-mixture baseline used diagonal covariance matrices, 100 expectation–maximization iterations, and a variance floor of 10^{-3}. The kernel-density sampler chose a stored PCA point uniformly and added Gaussian noise with the empirical PCA covariance and Scott bandwidth $h = K^{-1/(d+4)}$; $10^{-8}\mathbf{I}$ was added for numerical factorization. The nearest-neighbor sampler chose a stored point, selected one of its three nearest neighbors uniformly, and interpolated between them with a coefficient drawn from Uniform$(0, 1)$. The Gaussian copula transformed empirical marginal ranks to normal scores, estimated their correlation matrix with $10^{-6}\mathbf{I}$ added for factorization, and mapped sampled scores back through the empirical marginal quantiles. All samples were passed through the same inverse-PCA and destandardization pipeline and evaluated with the same marginal, cross-visit, mechanistic, and novelty calculations.

Selecting the inverse-temperature operating point

For each candidate β, we computed the Shannon entropy of softmax$(\beta\,\hat{\mathbf{X}}^{\top}\boldsymbol{\xi})$ at 20 stored-pattern probes, averaged the values, and divided by $\log K$. Zero means one pattern carries all attention; one means all patterns have equal attention. We evaluated 80 logarithmically spaced values from 0.1 to 1000 and selected β^* at the most negative finite-difference second derivative with respect to $\log\beta$. This marked the shift from shared to concentrated attention. For conditioned sampling, we repeated the calculation with the $\log r_k$ bias, producing $\beta^*(\rho)$. The random-pattern scale $\sqrt{d}$ was the theoretical reference.

Supplementary Tables

Table S1: **Inverse-temperature sensitivity.** Generation metrics across β/β^*, where $\beta^* \approx 2.94$ was selected from the attention-entropy transition in the 18-dimensional PCA space. The bold row marks the reported value. Med MRE is the median relative error of feature means; novelty is $1 - \max_k \cos(\hat{\xi}, \hat{m}_k)$.

β/β^*	β	Noise scale	PC1 std ratio	Novelty	Med MRE (%)
0.1	0.29	0.261	0.538	0.542	0.7
0.3	0.88	0.151	0.550	0.536	0.7
0.5	1.47	0.117	0.562	0.528	0.7
**1.0**	**2.94**	**0.082**	**0.589**	**0.502**	**0.6**
1.5	4.41	0.067	0.613	0.474	0.7
2.0	5.88	0.058	0.638	0.446	0.7
3.0	8.82	0.048	0.689	0.403	0.8

Table S2: **Multiplicity weighting parameters for conditional generation.** For the unit-normalized memories used here, the multiplicity ratio ρ was computed to place a target fraction $f_{\text{target}} = 0.80$ of finite-mixture component probability on the designated patterns. K_{eff} is the participation ratio of those component probabilities and describes their concentration across repeated draws.

Condition	K_{sub}	K_{bg}	ρ	f_{eff}	K_{eff}
Uncomplicated	18	5	1.11	0.80	23.0
PCOS	3	20	26.67	0.80	4.6
Developed PE	5	18	14.40	0.80	7.7

Table S3: **SA generation hyperparameters.** The same values were used for unconditioned and conditioned generation except that β^* was recomputed with the multiplicity bias for conditioned cohorts.

Parameter	Value	Description
α	0.01	Langevin step size (see Table S8)
T	2,000	Langevin iterations per sample
β^*	2.94	Entropy-transition point (unconditioned)
PCA threshold	95%	Variance retained
d_{PCA}	18	PCA dimensionality
N	100	Synthetic patients per cohort
Random seed	42	For reproducibility
f_{target}	0.80	Designated-component probability (conditioned)

Table S4: **BZ2012 ODE model calibration.** Five rate constants were calibrated on Visit 1 real patients (n=23) using bounded Nelder–Mead optimization in log-scale-factor space. The cost function was the mean normalized relative squared error across five TGA features under TF-only conditions. Bounds: $\pm\log(100)$. Convergence: $f_{\text{tol}} = 10^{-6}$, 500 iterations, 11 random restarts.

Rate constant	Role	Default	Calibrated	Scale
prothrombinase k_{cat}	Thrombin burst	63.5 s^{-1}	21.94 s^{-1}	0.35×
intrinsic Xase k_{cat}	Amplification Xa	8.2 s^{-1}	0.169 s^{-1}	0.021×
extrinsic Xase k_{cat}	Initiation Xa	6.0 s^{-1}	93.2 s^{-1}	15.5×
PC activation k_{cat}	Protein C activation	0.41 s^{-1}	0.890 s^{-1}	2.17×
mIIa conversion k	mIIa→IIa	2.3×10^8	1.15×10^9	5.01×

Units for mIIa conversion: $M^{-1}s^{-1}$

Table S5: **Summary of mean relative errors across all 72 features.** Per-visit distribution of MREs between real and SA-generated synthetic patient means. The full feature-by-feature table (72 features × 3 visits = 216 entries) is provided in the code repository as `paper_summary_statistics.csv`.

Visit	Median	Mean	25th pct.	75th pct.	Maximum	MRE below 5%
V1 (baseline)	0.022	0.029	0.008	0.039	0.158	59/72 (81.9%)
V2 (1st tri)	0.009	0.021	0.004	0.022	0.224	65/72 (90.3%)
V3 (3rd tri)	0.011	0.016	0.005	0.024	0.062	69/72 (95.8%)
**Pooled (all 3 visits)**	**0.012**	**0.022**	**0.005**	**0.028**	**0.224**	**193/216 (89.4%)**

Pooled median MRE 95% bootstrap CI: (0.98%, 1.60%) over 10,000 resamples (seed 42).

Table S6: **Comparison with deep generative baselines.** CTGAN and TVAE were trained on the same 90 per-visit records (23 patients × 3 visits, 72 features) at three epoch counts. Results for SA and MVN are shown for reference. The CTGAN model had median MRE near 19%, consistent with mode collapse at this sample size. The TVAE model matched marginal fidelity at high epoch counts but could not capture cross-visit covariance or perform conditional generation.

Method	Epochs	Med MRE	Med KS	Corr MAE
SA	–	0.019	0.169	0.200
MVN	–	0.024	0.161	0.090
CTGAN	300	0.189	0.364	0.261
CTGAN	1,000	0.195	0.349	0.265
CTGAN	3,000	0.187	0.341	0.180
TVAE	300	0.037	0.174	0.152
TVAE	1,000	0.026	0.248	0.082
TVAE	3,000	0.018	0.224	0.092

Table S7: **PCA variance threshold sensitivity analysis.** SA generation quality is stable across thresholds from 85% to 99%. Median MRE remains between 1.0% and 1.7% across all thresholds. The 95% threshold used in the main text (bold) is not a critical design choice.

Threshold	d_{PCA}	K/d	β^*	Novelty	Med MRE	Corr MAE
85%	13	1.77	2.94	0.420	0.0098	0.124
90%	15	1.53	2.94	0.477	0.0160	0.126
**95%**	**18**	**1.28**	**2.94**	**0.500**	**0.0124**	**0.143**
97.5%	20	1.15	2.94	0.543	0.0120	0.135
99%	21	1.10	2.94	0.541	0.0172	0.138

Table S8: **ULA and MALA sampling diagnostics.** Ten chains of 5,000 iterations each were run with both the Unadjusted Langevin Algorithm (ULA) and the Metropolis-Adjusted Langevin Algorithm (MALA) at step size $\alpha = 0.01$ and $\beta^* = 2.94$ on the 18-dimensional PCA memory (K=23 patterns). The MALA acceptance rate was 99.9%, and the energy and effective sample-size estimates were similar between methods, indicating little discretization bias. This comparison bounded the discretization error of the reported sampler. The direct reference for the target itself was the analytic sampler of Eq. (3), introduced below.

Metric	ULA	MALA
Acceptance rate (%)	– (no rejection)	99.9 ± 0.03
Mean energy (post-burn-in)	1.932 ± 0.141	1.886 ± 0.231
τ_{int}	109.5 ± 49.5	106.7 ± 30.4
Effective sample size	41.8 ± 14.2	40.0 ± 10.2

Burn-in: 1,000 iterations discarded. τ_{int}: integrated autocorrelation time estimated with a windowed estimator (threshold 0.05). ESS = $(T - T_{\text{burn}})/\tau_{\text{int}}$.

Table S9: **Proximity to stored profiles.** Two measures compared synthetic and stored profiles. Novelty is the angular distance between a synthetic profile and its nearest stored pattern at $\beta = \beta^*$. Nearest-neighbor distances are computed in the per-visit standardized 72-dimensional feature space.

Metric	Value
Mean novelty score, $1 - \max_k \cos(\hat{\xi}, \hat{m}_k)$	0.50
Median synth–real nearest-neighbor distance (std. units)	6.14
Median real–real nearest-neighbor distance (std. units)	6.87
Distance ratio (synth–real / real–real)	0.89

Table S10: **Descriptive bootstrap Mann–Whitney diagnostic per feature and condition.** For each of 24 feature–condition pairs, we subsampled the synthetic cohort to match the real sample size, applied a Mann–Whitney U test, and repeated 1,000 times. Frac. $p > 0.05$ is the fraction of replicates in which the test detected no difference at the available sample size. This power-limited diagnostic is not an equivalence test. Twenty of 24 pairs had $p > 0.05$ in $\geq 90\%$ of replicates; the four below-threshold pairs (italicized) involved high-variance features in the smallest subgroups.

Condition	Feature	MRE	Frac. $p > 0.05$
Uncomplicated (n=18)	FVIII	0.057	0.952
Uncomplicated (n=18)	vWF	0.022	0.999
Uncomplicated (n=18)	FIX	0.018	0.998
Uncomplicated (n=18)	FV	0.006	0.996
Uncomplicated (n=18)	α_2-AP	0.019	1.000
Uncomplicated (n=18)	FVII	0.057	0.941
Uncomplicated (n=18)	Fbgn	0.017	0.987
Uncomplicated (n=18)	Plgn	0.020	0.988
PCOS (n=3)	FVIII	0.109	0.985
PCOS (n=3)	vWF	0.150	0.959
PCOS (n=3)	*FIX*	*0.121*	*0.873*
PCOS (n=3)	FV	0.057	0.944
PCOS (n=3)	α_2-AP	0.051	0.987
PCOS (n=3)	FVII	0.029	0.999
PCOS (n=3)	Fbgn	0.043	0.992
PCOS (n=3)	*Plgn*	*0.153*	*0.615*
Developed PE (n=5)	FVIII	0.097	0.932
Developed PE (n=5)	vWF	0.010	0.986
Developed PE (n=5)	*FIX*	*0.108*	*0.780*
Developed PE (n=5)	FV	0.006	0.987
Developed PE (n=5)	α_2-AP	0.062	0.950
Developed PE (n=5)	FVII	0.045	0.993
Developed PE (n=5)	Fbgn	0.048	0.996
Developed PE (n=5)	*Plgn*	*0.093*	*0.814*

Median Frac. $p > 0.05$ across all 24 pairs: 0.986. Failing pairs (< 0.90, italicized): PCOS–FIX, PCOS–Plgn, DPE–FIX, DPE–Plgn.

Table S11: **Mechanistic validation diagnostics per feature and condition.** Cloud overlap is the fraction of synthetic ODE-predicted/measured ratios falling within the 5th–95th percentile of the corresponding real distribution. KS D and KS p are from a two-sample Kolmogorov–Smirnov test on the same ratio distributions. Real n=69 (23 patients × 3 visits); synthetic n=300 (100 patients × 3 visits).

Condition	TGA Feature	Cloud overlap	KS D	KS p
TF-only	Lagtime	0.92	0.112	0.48
TF-only	Peak	0.91	0.081	0.86
TF-only	T.Peak	0.92	0.102	0.61
TF-only	Max Rate	0.91	0.083	0.84
TF-only	ETP	0.83	0.123	0.36
TF+TM	Lagtime	0.92	0.127	0.32
TF+TM	Peak	0.88	0.079	0.88
TF+TM	T.Peak	0.91	0.110	0.51
TF+TM	Max Rate	0.88	0.096	0.67
TF+TM	ETP	0.89	0.112	0.48

Under TF-only conditions, the cloud overlap range was 0.83–0.92. Under TF+TM conditions, the range was 0.88–0.92. Pooled across both conditions and all five features, the overlap was 0.902. KS D range across both conditions: 0.079–0.127, all $p > 0.30$.

Table S12: **Downstream utility: per-feature held-out V2/V3 performance.** Median relative error of the BZ2012 ODE-predicted TGA values against the held-out real V2 and V3 measurements, for the model calibrated on real V1 patients (K=23) and the model calibrated on synthetic V1 patients (N=100). The synth-calibrated model achieves slightly lower error on every feature. Synth/Real ratio < 1 favors the synth-calibrated model.

TGA Feature	Real-cal MRE	Synth-cal MRE	Synth/Real
Lagtime	0.165	0.162	0.98×
Peak	0.097	0.087	0.90×
T.Peak	0.330	0.306	0.93×
Max Rate	0.286	0.259	0.91×
ETP	0.197	0.183	0.93×
**Overall median**	**0.201**	**0.189**	**0.94×**

Table S13: **PCA-space ablation.** Five generators used the same d=18 PCA subspace and evaluation. PCA+GMM is the fitted two-component diagonal-covariance baseline, not the exact stochastic-attention mixture. MRE measures marginal fidelity. Mechanistic overlap is the fraction of pooled synthetic predicted-to-recorded TGA ratios within the real 5th–95th percentile range. Median novelty is $1 - \max_k \cos(\hat{\boldsymbol{\xi}}, \hat{\mathbf{m}}_k)$; Frac. novel is the fraction above 0.2. Cross-visit Frobenius error measures deviation from the real correlation matrix. Lower error indicates closer in-sample reproduction and can reward memorization.

Method	MRE (%)	Cross-visit Frob.	Mech. overlap	Median novelty	Frac. novel
SA	1.24	0.641	0.902	0.514	1.00
PCA+GMM	1.13	0.422	0.918	0.497	0.94
PCA+KDE	1.94	0.337	0.823	0.249	0.65
PCA+kNN	1.63	0.587	0.938	0.051	0.05
PCA+Copula	1.34	0.417	0.934	0.462	1.00

Table S14: **Convex-hull geometry and plausibility at K=23 in d=18.** We compared 100 synthetic profiles with leave-one-out real profiles using hulls of 22 real points. For each synthetic profile, we omitted its nearest real profile. The radial overshoot $1/t^*$ used the point where a ray from the hull centroid exited the hull. All profiles lay outside their comparison hulls. Median hull distance was 11.0 for synthetic profiles and 11.9 for real profiles (p=0.07); radial overshoot was smaller for synthetic profiles ($p < 10^{-4}$). These descriptive tests treated the synthetic profiles as independent, so their significance was optimistic. Hull membership had little resolution because every real profile formed an outer corner, and a unit-normalized generated direction could remain inside only by matching a stored direction. The biological check required nonnegative values and limited coagulation-factor and TGA values to five real-cohort standard deviations from the mean. Fifty-four synthetic profiles passed. The other 46 contained negative estradiol or progesterone values: estradiol was negative in 19 profiles and progesterone in 37, with 10 overlapping; the minima were −2109.8 and −75.8. No variable was constrained to be positive, and linear decoding had unbounded support. Hormones were closer to zero relative to their observed variation than the coagulation factors, which explained why only hormones crossed zero in these draws but did not guarantee positive factors in other draws. All nine factor inputs to BZ2012 were positive, so all 100 profiles entered the mechanistic check. The unconstrained MVN baseline produced negative hormones in 43 profiles, compared with 46 for stochastic attention.

Quantity	Synthetic (n=100)	Real, leave-one-out (n=23)
Fraction inside hull	0.00	0.00
Median distance to hull	11.0	11.9
Median radial overshoot $1/t^*$	10.5	15.2
Hull geometry of the real cohort		
Median patient radius	13.8	
Median hull extent, generic direction	2.0	
Largest t^* attained on the unit sphere	0.231	
Plausibility of the out-of-hull synthetic patients		
Biological constraints satisfied	54/100	
Mechanistic band, per feature	83 to 92%	
Mechanistic band, all features and visits	41/100	
Both biological and strict mechanistic criteria	24/100	

Table S15: **Membership inference recovered memory membership, not unseen patients.** Median nearest-neighbor distances in the standardized 216-dimensional space, and the test's discrimination. For each split, we constructed the memory matrix from 15 patients and scored each real record by its distance to the resulting synthetic set, treating the 15 included patients as members and the 8 held-out patients as non-members. Every quantity came from the same protocol. We drew 40 partitions at random with the generation seed held fixed, so that only the partition varied, and pooled the distances across all partitions. Non-members sat at the same distance from the synthetic cohort as unrelated real patients sat from one another. The synthetic cohort carried no proximity signal about patients the generator never saw, and the discrimination came entirely from members sitting closer. The area under the receiver operating characteristic curve (AUC), calculated using the equivalent Mann–Whitney rank statistic, measured how often the test assigned stronger membership evidence to a randomly chosen member than to a randomly chosen non-member; 0.5 indicated no discrimination and 1.0 indicated perfect discrimination.

Quantity	Value
Median nearest-neighbor distance	
Memory-matrix member to nearest synthetic	10.7
Held-out non-member to nearest synthetic	15.4
Real to nearest real, leave-one-out	15.9
Synthetic to nearest real	13.8
Membership-inference AUC over 40 *random splits*	
Mean	0.97
Standard deviation	0.02
Median	0.98
Minimum	0.90
Maximum	1.00
Splits reaching perfect separation	9/40
Splits with AUC above 0.9	39/40

Supplementary Figures

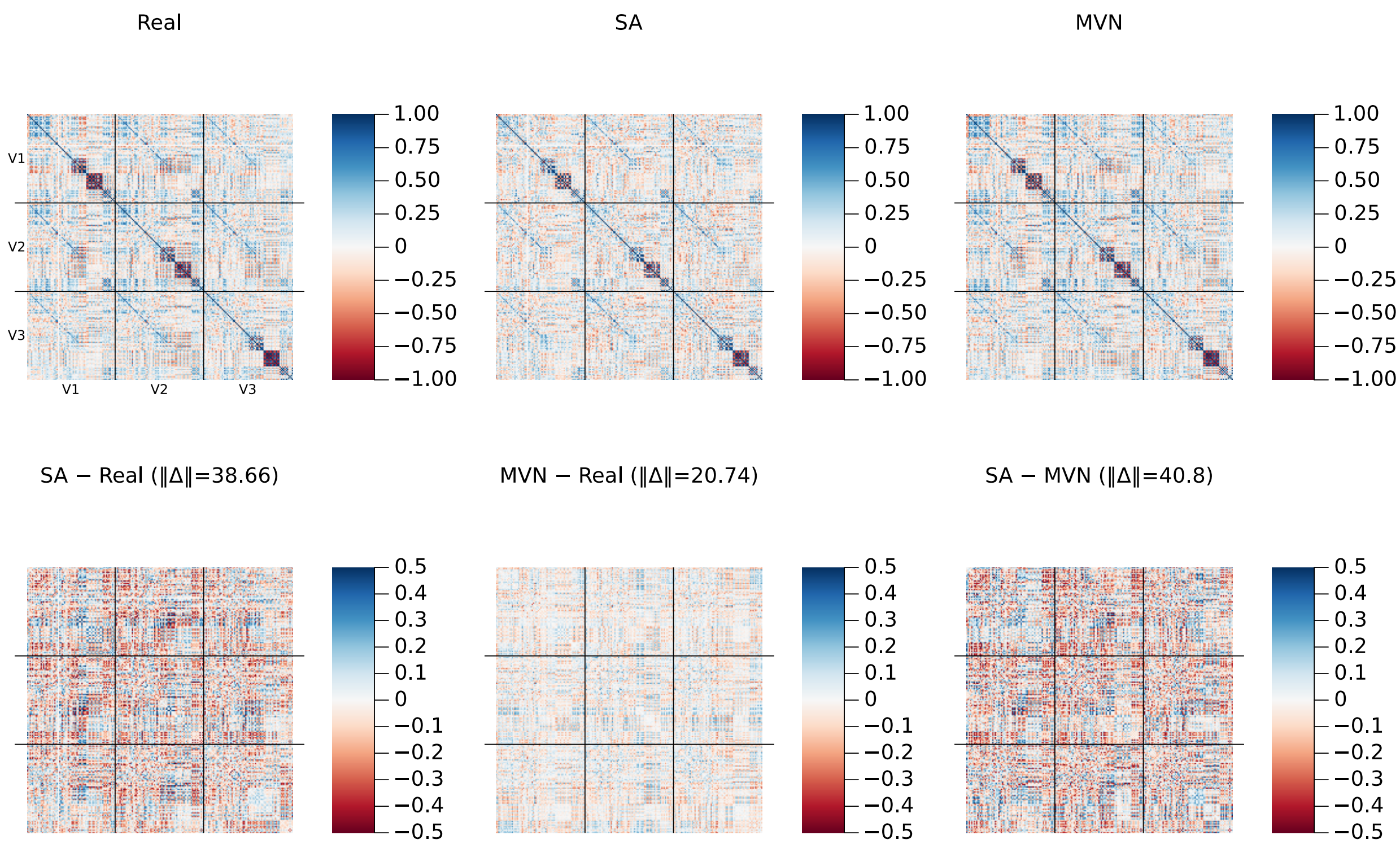

Figure S1: **Cross-visit correlation structure (amplified residual scale).** Same layout as the main-text correlation figure, but with the bottom-row residual panels plotted on a ± 0.5 color scale (vs. ± 1.0 in the main text) to reveal fine structure. Frobenius norms of each residual matrix are shown in the panel titles.

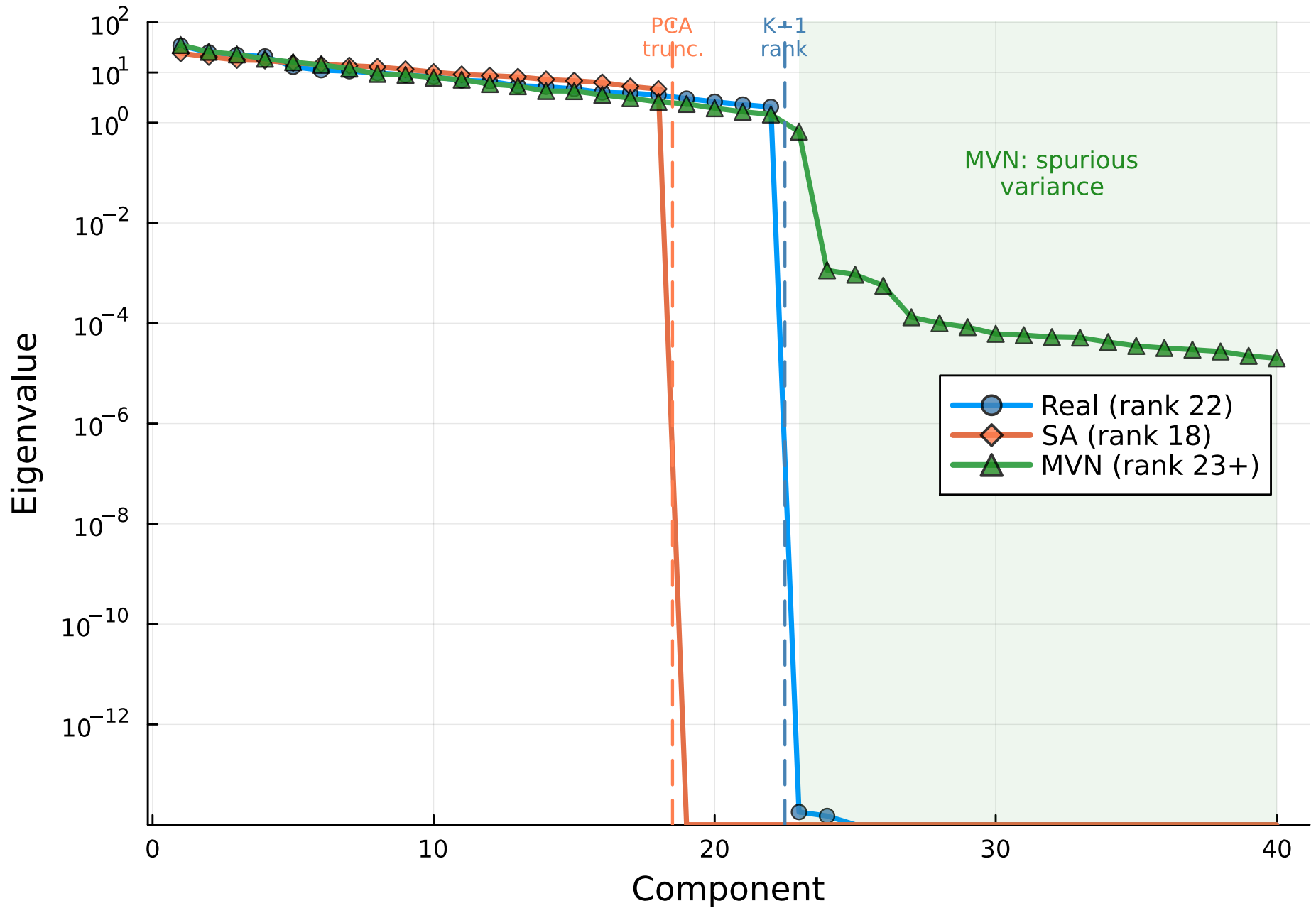

Figure S2: **Covariance eigenvalue spectrum.** Log-scale eigenvalues of the 216 × 216 sample covariance for real (K=23, rank 22), SA (N=100, rank 18), and MVN (N=100) populations. Dashed vertical lines mark the SA PCA truncation point (component 18) and the data rank limit (component 22). Ledoit–Wolf regularization for MVN inflates eigenvalues beyond component 22 (green shaded region), introducing spurious variance in 194 dimensions where the data contain no signal.

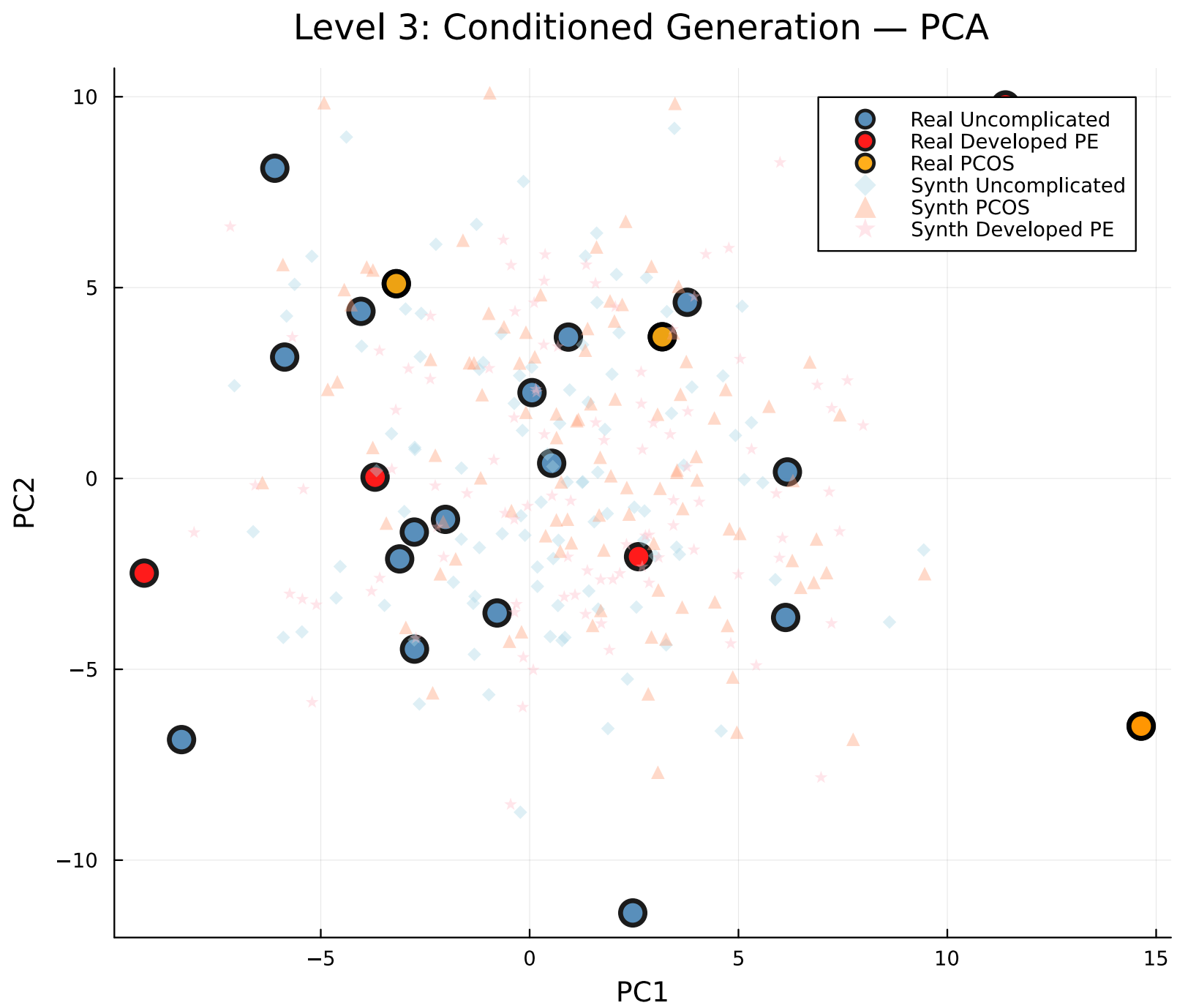

Figure S3: **Conditioned generation: PCA projection (pooled).** Large markers: real patients (blue = Uncomplicated, n=18; orange = PCOS, n=3; red = Developed PE, n=5). Small markers: synthetic patients (N=100 per condition). With only 3 PCOS and 5 Developed PE patients, the subgroups do not form clearly separable clusters in the first two principal components, but the synthetic cohorts concentrate around their respective real counterparts.

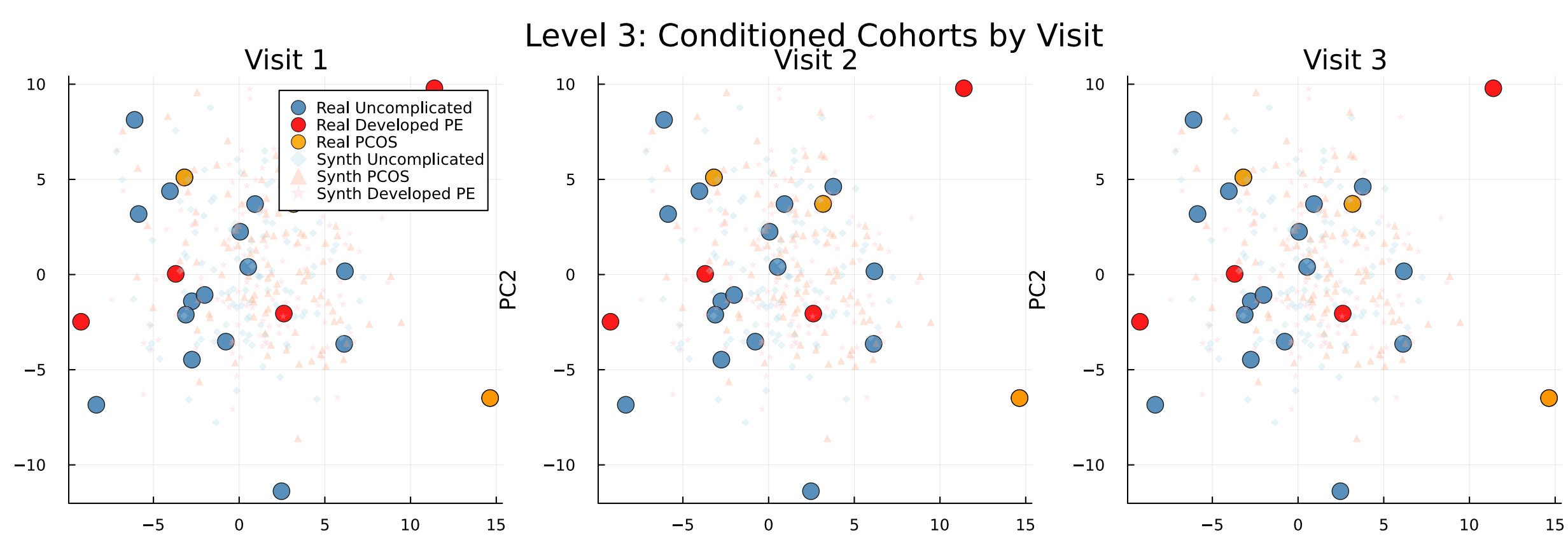

Figure S4: **Conditioned generation: PCA projection by visit.** Same as Fig. S3 but separated by visit (V1, V2, V3). Condition-specific clustering is maintained within each individual visit.

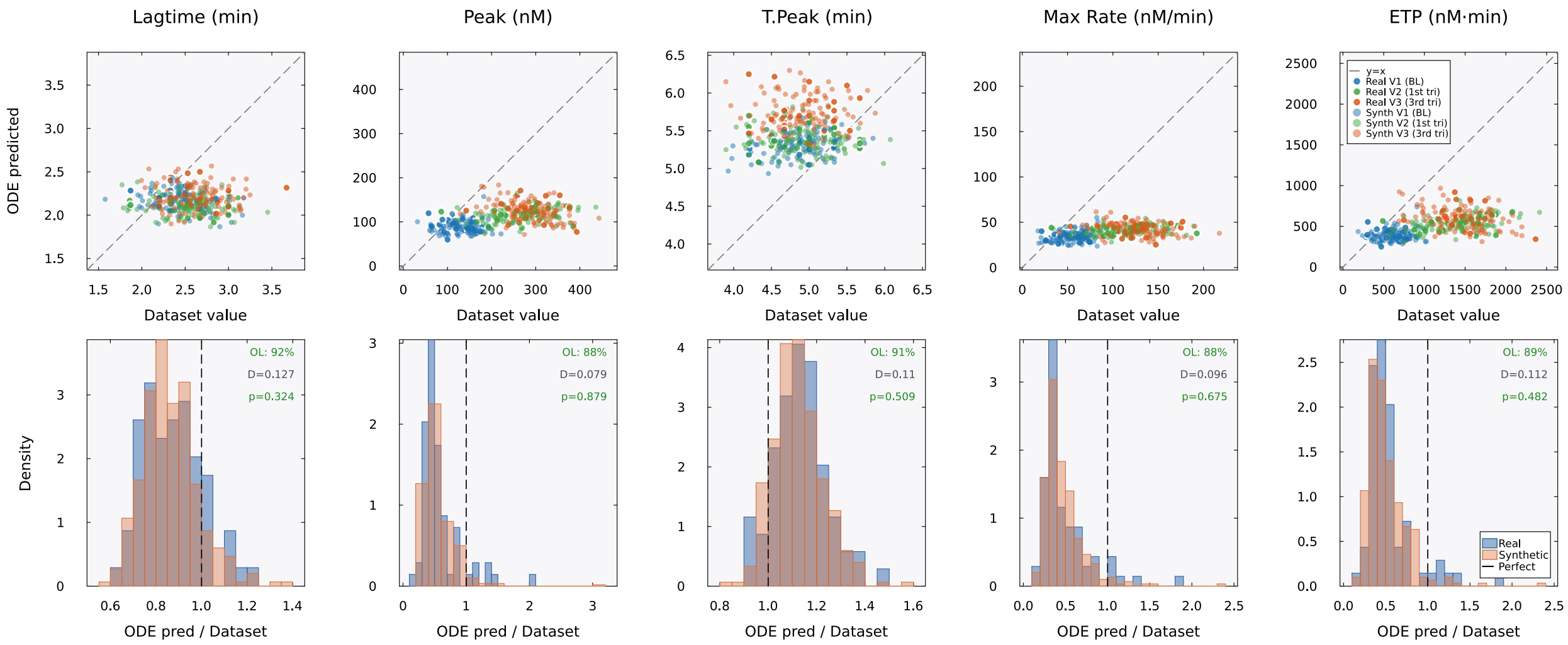

Figure S5: **Mechanistic validation (TF+TM condition).** Same layout as main-text Fig. 6 but under TF+TM conditions. The model systematically underpredicts peak and maximum rate ($\approx 0.5\times$ measured) for both real and synthetic patients, reflecting overcorrection of the protein C anticoagulant pathway. Cloud overlap was 88–92%, showing similar model behavior for real and synthetic profiles despite imperfect calibration.

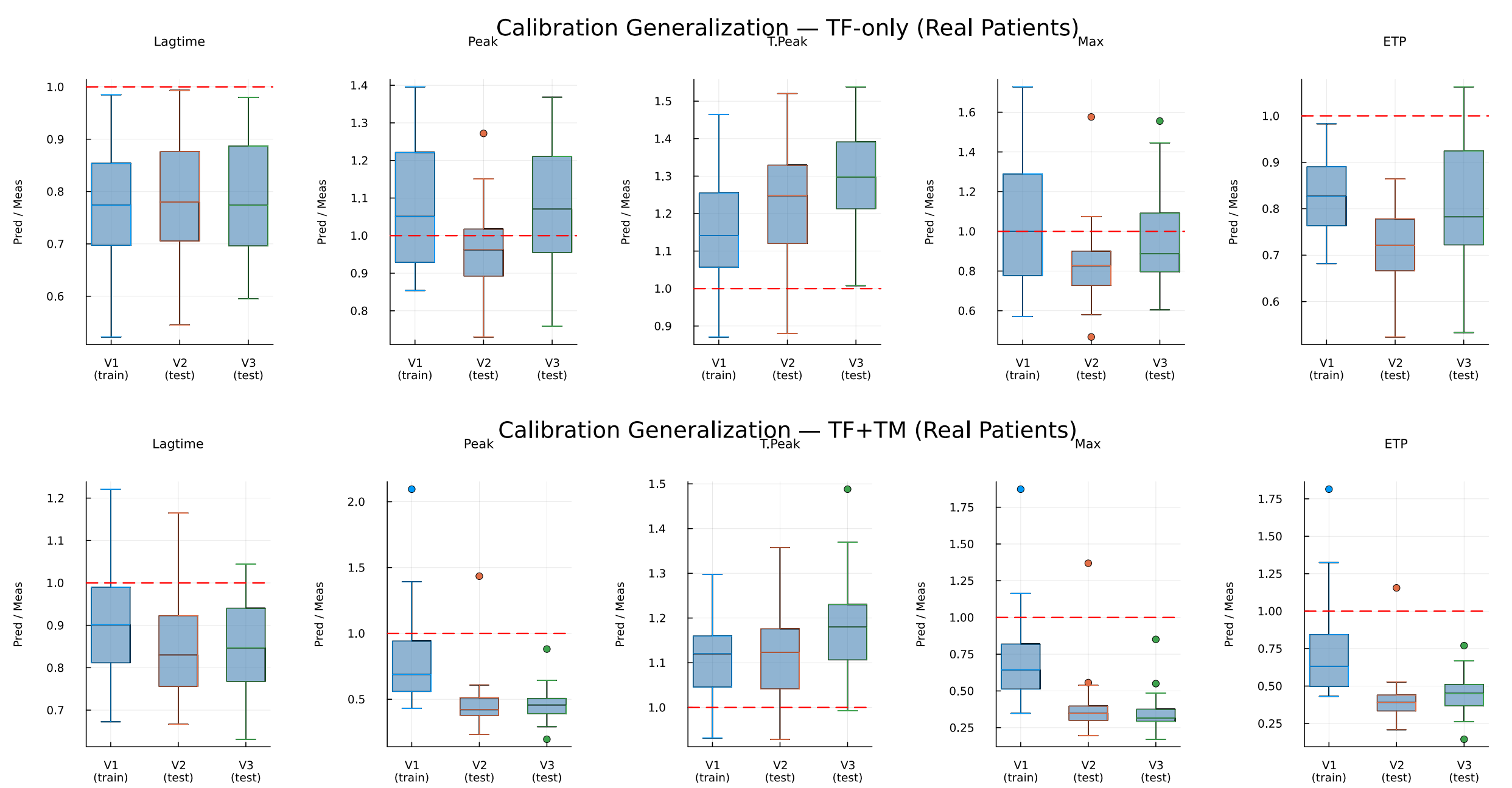

Figure S6: **Cross-visit calibration generalization.** Boxplots of the ODE-predicted/dataset ratio by visit for real patients. The model was calibrated on Visit 1 only. Top: under TF-only conditions, ratios remained near 1.0 at Visits 2 and 3. Bottom: under TF+TM conditions, the ratios differed more across visits, particularly for peak and maximum rate.

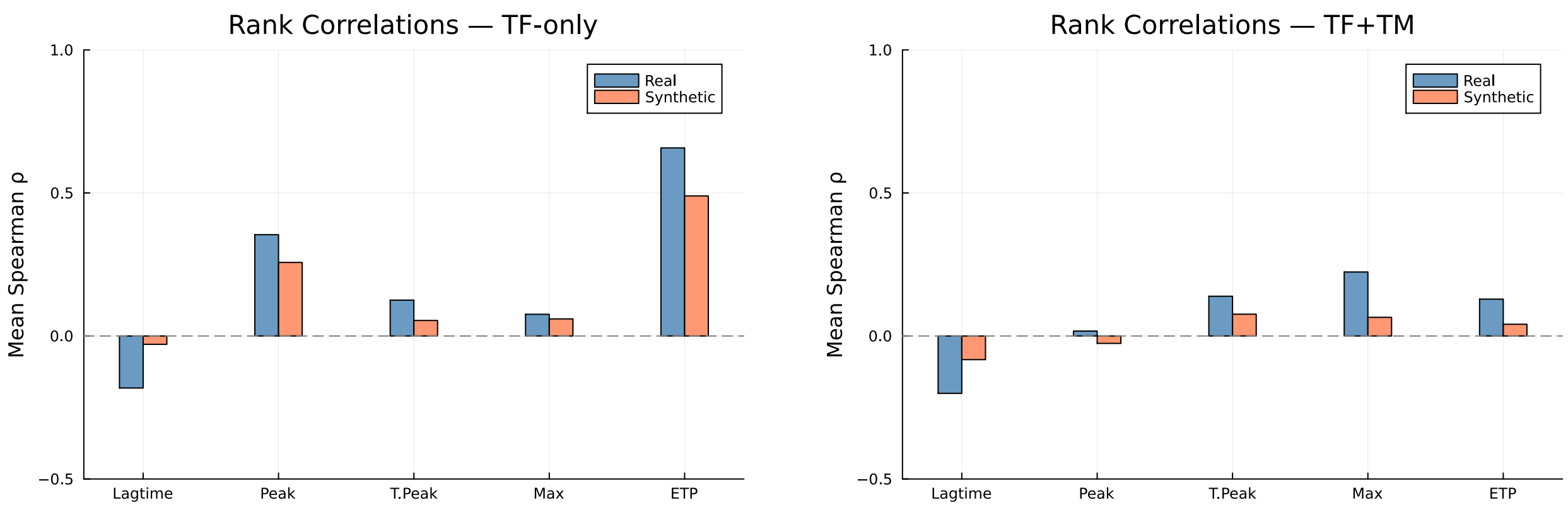

Figure S7: **Spearman rank correlations between ODE-predicted and dataset TGA features.** Left: TF-only. Right: TF+TM. ETP is the best-predicted feature ($\rho \approx 0.6$–0.8 for real patients under TF-only). Weak rank correlations for other features are expected given the 5-parameter population-level calibration.

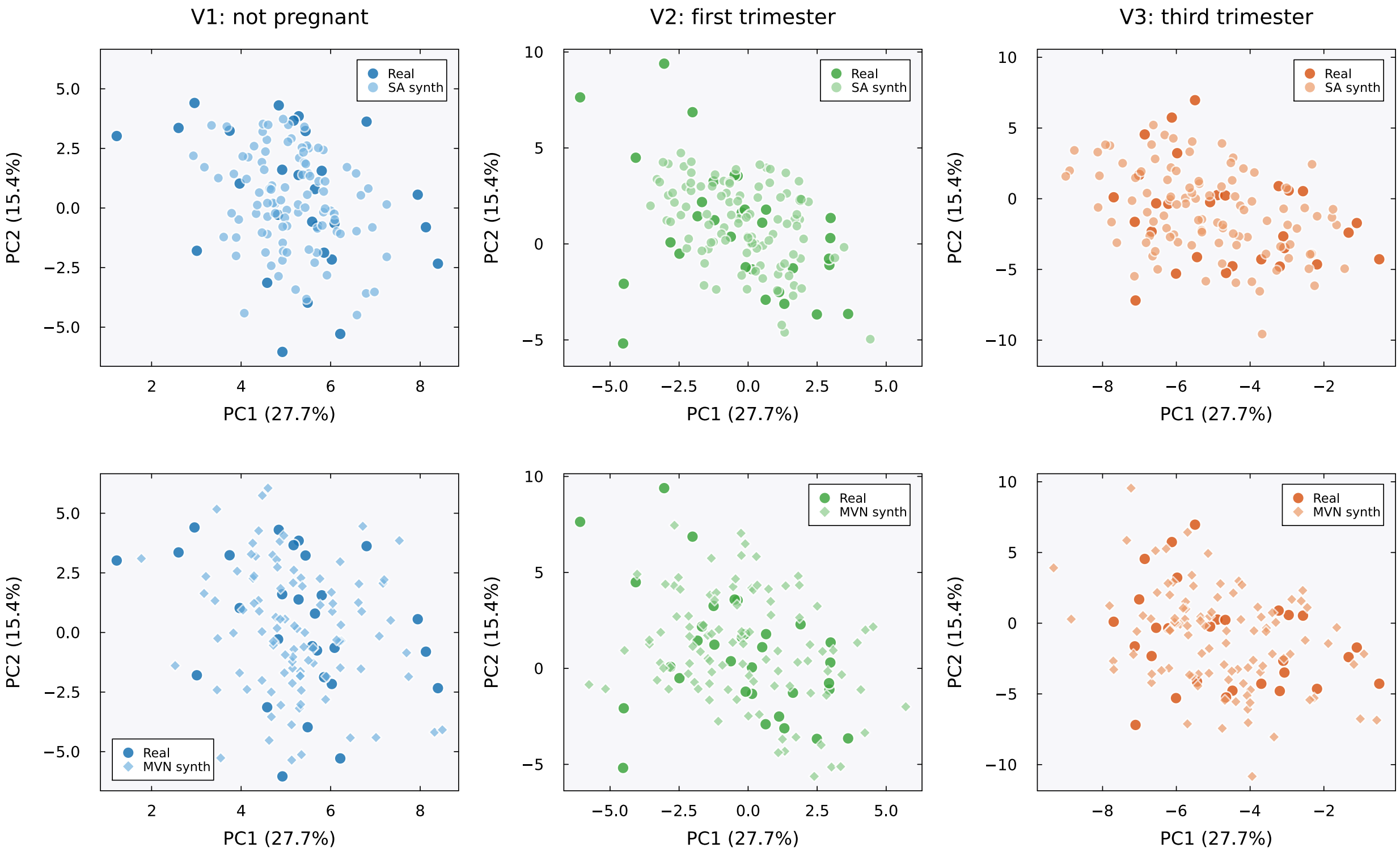

Figure S8: **PCA projections by visit: SA and MVN.** Each panel shows the first two principal components (PC1: 27.7% variance, PC2: 15.4%) computed from standardized per-visit real patient data (K=23 patients per visit, 72 features). Dark markers indicate real patients; lighter markers indicate synthetic patients (N=100). Within each visit (column), the SA panel (top) and MVN panel (bottom) share identical axis ranges, so the relative dispersion of the two methods can be read against the same real data cloud. Top row: SA-generated patients (circles) cluster tightly around the real data cloud at all three visits. Bottom row: MVN-generated patients (diamonds) show greater dispersion, particularly at Visits 2 (first trimester) and 3 (third trimester), where MVN patients extend beyond the convex hull of the real data. This excess scatter reflects the spurious variance introduced by Ledoit–Wolf regularization of the rank-deficient covariance (Fig. S2). Colors encode visit: blue (V1, not pregnant), green (V2, first trimester), orange (V3, third trimester).

Sampling correctness and privacy diagnostics

We asked whether a synthetic cohort could reveal that an already-known de-identified assay profile was stored in the memory matrix. The source data had no direct identifiers or links to named individuals, so this was not a test of personal identification. In standardized 216-dimensional space, the median distance to the closest real record (DCR) was 13.8 for synthetic profiles, compared with a leave-one-out real-to-real distance of 15.9. We then tested 40 random splits with 15 stored and 8 held-out profiles. Distance to the synthetic cohort distinguished the groups with mean AUC 0.97 (standard deviation 0.02; 0.5 meant no discrimination and 1.0 perfect discrimination). To test whether the numerical sampler caused this signal, we compared the reported sampler with sphere initialization, pooled samples after burn-in and thinning, and Metropolis-adjusted sampling (Table S16, Fig. S9). Metropolis acceptance was 0.999, and the AUC remained 0.96–0.97. Median DCR changed only from 14.0 to 14.3 and remained below 15.9. Changing the Markov-chain procedure therefore did not remove the membership signal.

We also varied the inverse temperature and sampled the target distribution directly. Across a 40-fold sweep around β^*, lower β increased median DCR but it plateaued near 14.3; cross-visit covariance error increased from 0.59 to 0.69 (Fig. S9C). The closed-form stochastic-attention target in Eq. (3) is the exact mixture $\sum_k q_k \mathcal{N}(\mathbf{m}_k, \beta^{-1}\mathbf{I})$, with $q_k = r_k / \sum_j r_j$ for unit-normalized memories. Its direct sampler requires only a component draw and isotropic Gaussian noise. It has no initialization, burn-in, or discretization error. A matched direct-sampler cohort gave median relative error 1.68%, cross-visit error 0.60, mechanistic overlap 0.91, median novelty 0.50, and median DCR 13.87, all within the range of the four Markov-chain procedures. Across 100 direct-sampler cohorts, median relative error was 1.40% (5th–95th percentile 0.97–1.91%) and median DCR was 13.95 (13.61–14.39). Exact sampling gave AUC 0.98 over the same 40 splits. This showed that the membership signal came from the target distribution, not from the numerical sampler. At this cohort size, the test could therefore distinguish whether an already-known de-identified profile was stored regardless of how the target distribution was sampled.

Table S16: **Sampler comparison.** All procedures used the same β^*, memory, and decoder. We report median relative error (MRE), cross-visit correlation error, mechanistic overlap, novelty, DCR, and membership-inference AUC where evaluated. The real-to-real DCR baseline was 15.9. The AUC used the same 40 splits for V0, V3, and the direct stochastic-attention sampler. *Exact* drew directly from Eq. (3) and was not a Markov-chain procedure. Across 100 direct-sampler cohorts, median MRE was 1.40% (5th–95th percentile 0.97–1.91%) and median DCR was 13.95 (13.61–14.39).

Rung	Sampler	MRE (%)	Cross-visit Frob.	Mech. overlap	Novelty	DCR	MIA AUC
V0	anchor, endpoint	1.31	0.65	0.89	0.51	13.97	0.97
V1	sphere init	1.53	0.57	0.89	0.50	13.96	n/a
V2	pooled ULA	2.02	0.59	0.88	0.50	14.15	n/a
V3	pooled MALA	1.89	0.62	0.92	0.52	14.33	0.96
Exact	analytic ancestral	1.68	0.60	0.91	0.50	13.87	0.98

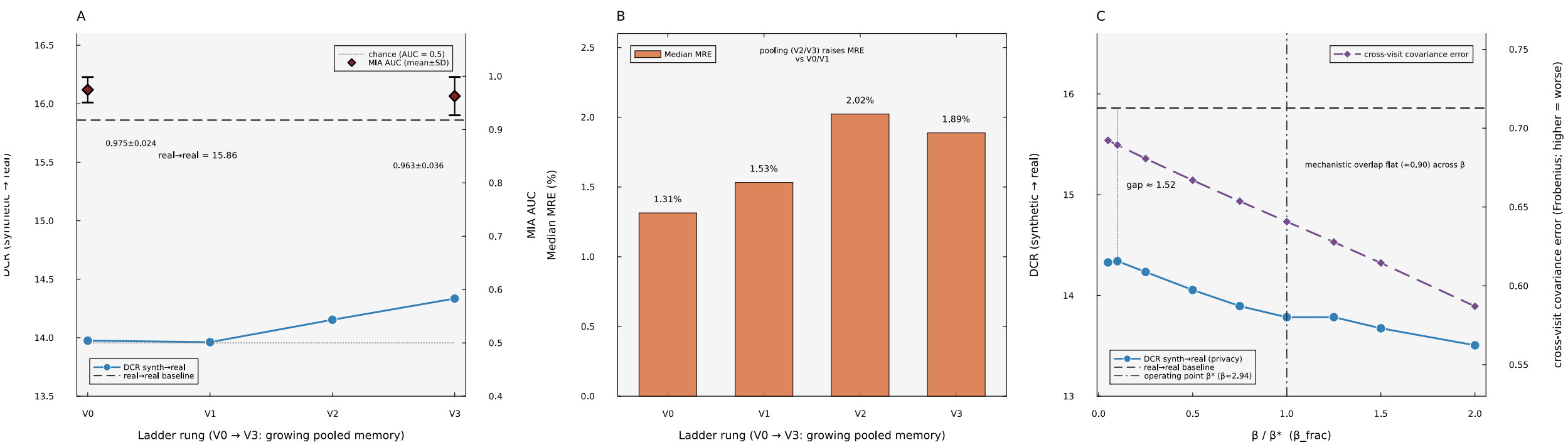

Figure S9: **Sampling correctness and the membership signal.** (A) Across V0–V3, DCR rose slightly but stayed below the real-to-real baseline, while membership-inference AUC remained 0.96–0.97. (B) Median relative error across the four procedures. (C) Across a 40-fold inverse-temperature sweep around β^*, DCR increased as β fell but remained below the real-to-real baseline. Cross-visit covariance error increased, while mechanistic overlap changed little.

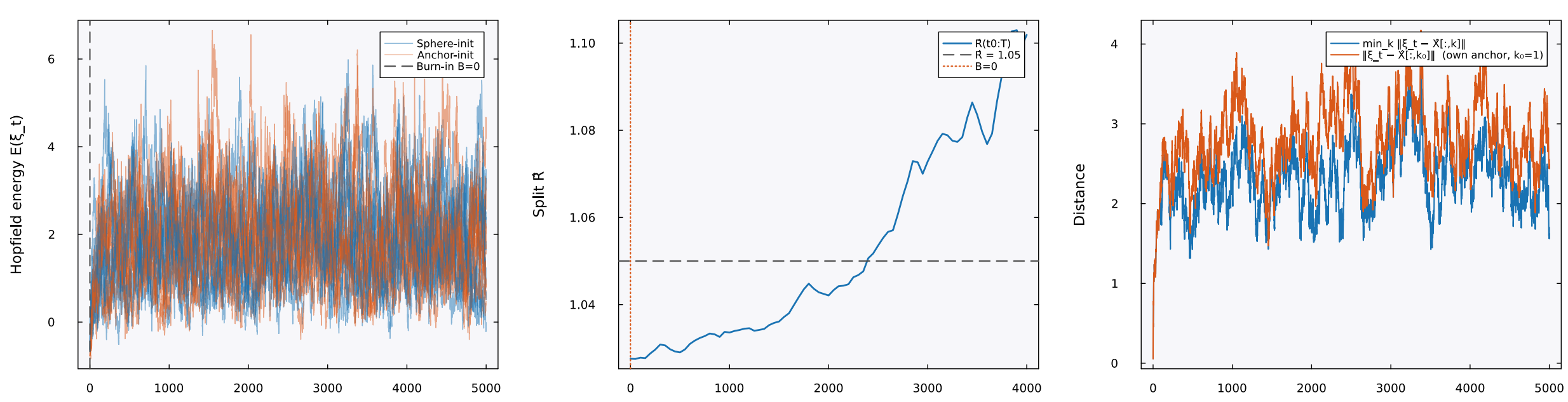

Figure S10: **Mixing diagnostic at β^*.** Energy traces, the between-chain $\hat{R}$ statistic, and the anchor-distance trajectory for sphere-initialized Langevin chains. The target distribution at β^* was multimodal, with one component centered on each stored pattern. Metropolis acceptance near 0.999 indicated negligible discretization bias.
